# Health systems strengthening in the Democratic Republic of Congo: the importance of surgical data

**DOI:** 10.1136/bmjgh-2024-017759

**Published:** 2025-09-04

**Authors:** Achim Mambu Vangu, Michel Nlandu Kambu, Joenel Mfundu Mbuangi, Andrew J M Leather, Elizabeth H Tissingh

**Affiliations:** 1 Hôpital Provincial de Référence Kinkanda, Matadi, Congo (the Democratic Republic of the); 2 King’s Global Health Partnerships, School of Life Course and Population Sciences, King’s College London, London, UK; 3 Division Provincial de la Santé, Matadi, Congo (the Democratic Republic of the); 4 Centre de Santé de Référence Luila, Luila, Congo (the Democratic Republic of the)

**Keywords:** Health systems, Health policy, Health systems evaluation, Surgery

## Abstract

World Health Assembly resolutions have recently placed emphasis on emergency, critical and operative care to achieve universal health coverage. This will require a move away from vertical health programmes and a stronger emphasis on health systems strengthening, including data systems. There has also been an increased recognition of the need for data to advance the global surgery agenda. We share our efforts in the Democratic Republic of Congo (DRC) to strengthen surgical data collection, analysis, interpretation and dissemination sustainably. Working with individuals, institutions and systems, we outline interventions used to strengthen the health system and lessons learnt. Our work has highlighted the negative consequences of vertical programmes with demanding data management processes and the importance of taking a systems-level approach to improving surgical data and ultimately surgical outcomes.

**SUMMARY BOX**

- There is increasing recognition that health systems strengthening, including data systems, is essential to achieve healthcare for all.
- We present programmatic work in the Democratic Republic of Congo that illustrates one approach to health systems strengthening.
- The partnership approach presented in this work may offer a useful approach for others engaged in health systems strengthening.

## Introduction

 World Health Assembly (WHA) resolutions (WHA 60.22,^1^ WHA 68.15,^2^ WHA 76.2^3^) have recently placed emphasis on emergency, critical and operative care to achieve universal health coverage (UHC). There has also been an increased recognition of the need for data to advance the global surgery agenda.^4^ For instance, the WHO Safe Surgery Saves Lives initiative^5^ had a pillar on surgical data, and the Lancet Commission on Global Surgery (LCoGS) recommended routine collection of six surgical indicators.^6^ However, the availability, comparability and utility of these indicators have been problematic,^7^ leading to a revision published as an Utstein Consensus report.^8^ (See online supplemental material 1 for summary on currently recommended indicators.)

Despite the recognition that surgical care is an essential part of UHC and the recognition that data and indicators to monitor progress are important, there are few examples of surgical indicators being used to evaluate systems or guide practice.^9^ We share our efforts in the Democratic Republic of Congo (DRC) to strengthen surgical data collection, analysis, interpretation and dissemination sustainably.

### Safe surgery programme in Kongo Central, Democratic Republic of Congo

**Figure 1:**
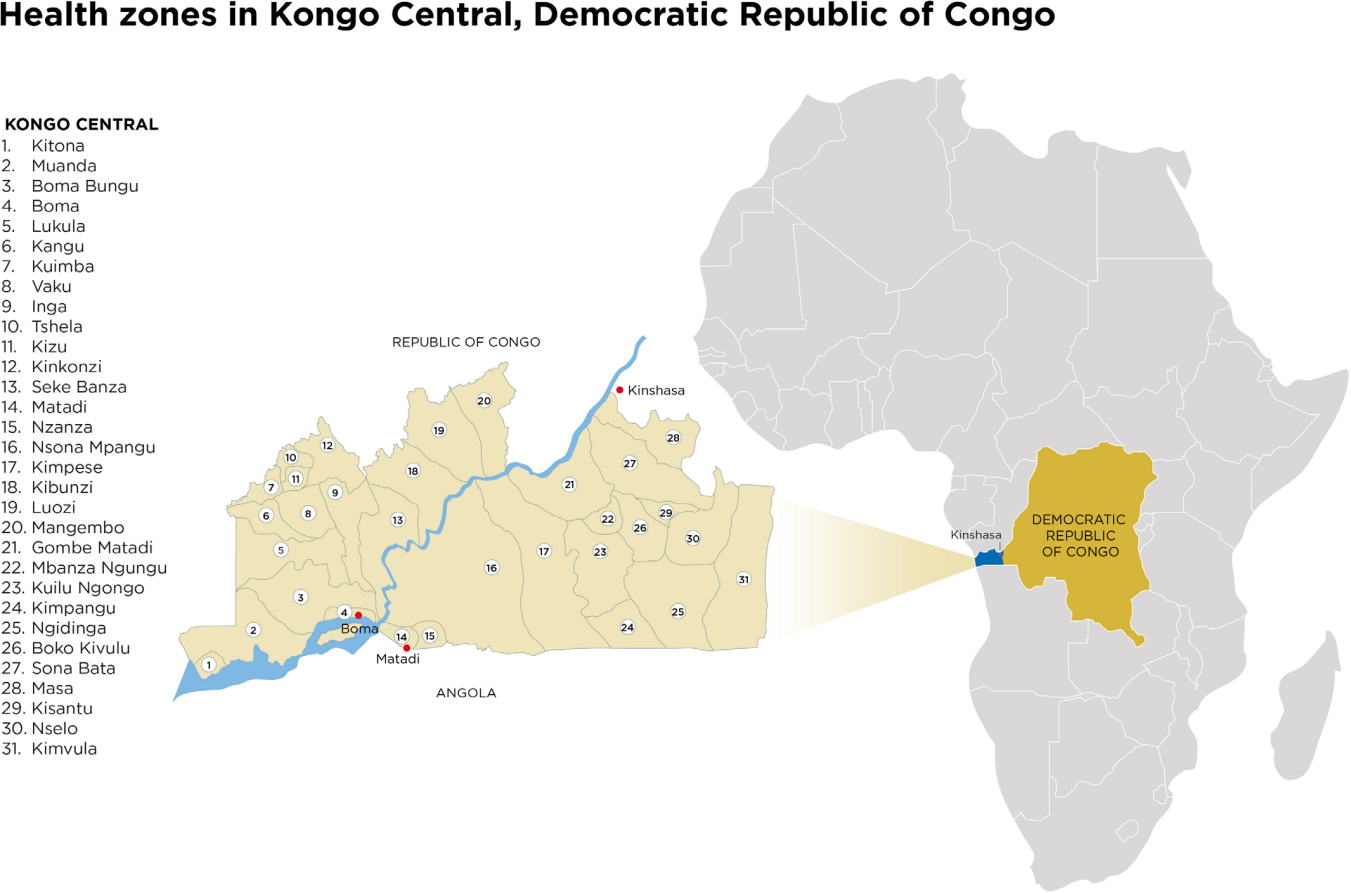
Democratic Republic of Congo in Central Africa, with Kongo Central province and 31 health zones highlighted.

King’s Global Health Partnerships has worked with the Kongo Central Provincial Ministry of Health, Division of Health, and the 31 health zones since 2015 (figure 1 and Explainer Box 1). Kongo Central is a stable but neglected province in the southwest of the DRC, with a population of approximately six million people. The Safe Surgery programme in Kongo Central began in 2018 and has built a community of practice using principles from the WHO Safe Surgery Saves Lives initiative.^5^ This seeks to improve the safety of surgical care by deﬁning a core set of safety standards, including patient monitoring with oxygen saturation probes; improving infection, prevention and control; and implementation of the WHO Safe Surgery Checklist.

**Table 1:** Programme partners

Ministère provincial de la santé (MdS) (Provincial Ministry of Health)	The Provincial Ministry of Health acts to oversee nationally set strategy. The minister is a political appointment and may not have a healthcare background. The health advisor working in the ministry is usually a doctor. Since 2013, there have been seven ministers.
Division provincial de la santé (Provincial Division of Health)	The Division of Healthcare is the technical delivery office of the Ministry of Health. The clinical director oversees several technical offices including the Bureau d’appui Technique (technical support office), Bureau INFOSAN (Health Information System) and the Bureau Ressources Humaines (Human Resources Office). The division is staffed by clinicians and experienced advisors and implements programmes such as mosquito bed net distributions, vaccination campaigns and led on the COVID-19 response.
Zones de santé (health zones)	Kongo Central has 31 zone de santé (health zones) each led by a Médecin chef de zone (head of the health zone, qualified doctor). Each health zone has a centre de santé (health centre) and a hôpital général (district level hospital); however, the size of these, and how well equipped they are, varies in each health zone. Some health zones also have a hôpital provincial de référence (tertiary referral hospital); there are five in Kongo Central.
King’s Global Health Partnerships	King’s Global Health Partnerships works with health facilities, academic institutions and governments to strengthen health systems and improve the quality of care in five countries: Gambia, Somaliland, Sierra Leone, Democratic Republic of Congo and Zambia. It brings together expertise from King’s College London, King’s Health Partners’ NHS Trusts, and our international partners to educate, train and support healthcare workers, strengthen healthcare and training institutions and enhance national health policies and systems.

A baseline assessment highlighted the challenges and opportunities for surgical care (online supplemental material 2). The four key findings were as follows: (1) there is a large unmet need for surgical care in Kongo Central, (2) the main types of surgery being conducted are caeserean section and trauma surgery, (3) there is a critical shortage of surgical specialists and (4) there is a critical shortage of infrastructure and equipment for surgery. To tackle these challenges, the theory of change for the programme (online supplemental material 3) has been developed over the years of collaborative working and outlines change at the micro, meso and macro levels to ensure that knowledgeable, trained and confident healthcare providers work in well-equipped facilities, all guided by good data and governance.

Earlier work highlighted the need for improved data, and the current phase of the programme, starting in 2022, placed greater emphasis on improving data collection, analysis, interpretation and dissemination across the province. A key component of the work was the collection of surgical data to monitor patient safety and for use in policy development; the details of the data workstream emerged as the work progressed. The key programme milestones are illustrated in online supplemental material 4, which highlights the iterative nature of the work carried out.

### Understanding the data landscape

Three main healthcare data sets capture surgical data in Kongo Central. Firstly, facility-level theatre logbooks collect surgical data, and ward logbooks collect data related to postoperative outcomes. Secondly, the National Health Information System (Système National d’Information Sanitaire (SNIS)) collects general health data at the facility level. Thirdly, the District Health Information Software 2 (DHIS2) compiles health data at the provincial level, with the data input into the SNIS tools. To better understand data collection, input and analysis, a situational analysis was conducted from March 2022 to April 2023. This included direct observation during hospital site visits at six secondary and four tertiary healthcare facilities and interviews at the first senior stakeholder workshop. As the work progressed, we thought a fuller understanding of healthcare workers’ perceptions about data would be helpful, and a survey tool was developed and administered. A situational analysis report, available both in French and English, with detailed methodology and findings, was published online (online supplemental material 5).

### Implementing change: multifaceted interventions

Through the review of the situational analysis and regular contact between stakeholders, including the project implementation team, the work was developed with a multifaceted approach. These interventions are summarised in Explainer table 2 and then discussed in detail.

**Table 2:** Interventions in the data workstream

Intervention	Time frame	Explanation
Senior stakeholder workshops	September 2022 to February 2024	The first workshop brought together key stakeholders and started the intervention design. The second workshop, towards the programme end, reviewed progress and ensured sustainable implementation of the interventions.
Data working group	Begun September 2023	A working group, with involvement of more members of the DPS team, was established 12 months into the work.
Training	Safe surgery and quality improvement methodology course: September 2022 to January 2023 Data training: February 2023	The safe surgery and quality improvement methodology course, with a module on data, was delivered to all 10 sites using a train-the-trainers approach. In addition, targeted training on data collection was delivered.
New indicators	September 2022 to March 2023	The paper ‘Canevas supplémentaire’ was piloted first, and following review, new indicators agreed with electronic data collection.
Quarterly site visits	September 2022 to present	Visits, led by the DPS, to all 10 sites allowed for the review of data collection, troubleshooting and shared learning.
National engagement	February 2024	This work was presented at the national level, with a view to incorporating this approach into the National Quality of Care Strategy.

DPS, Division provinciales de la santé.

### First senior stakeholder workshop

A stakeholder workshop was held in September 2022, with senior leadership from the provincial ministry and divisional offices as well as clinicians from the health zones and healthcare facilities. Surgical data challenges and opportunities were identified. The findings from the baseline hospital site visits and interviews were presented and discussed. A presentation was given on surgical indicators that the global community was advocating, and a data collection tool (‘canevas supplémentaire’) was developed during the workshop that was informed by the situational analysis, international recommendations and stakeholder reflections on feasibility (online supplemental material 6). This data collection tool was subsequently piloted by health zones, and feedback was given to DPS leadership. The piloting process was heterogeneous, and uptake of the additional tool was patchy.

### Working group

The overall programme was led from the beginning by a project implementation team, which included surgeons from the four main referral hospitals and key members within the DPS. This team led on the data workstream as part of the overall programme of work. However, in view of the importance of surgical data and the synergy between surgical metrics and quality of care, a larger working group with a detailed workplan was set up in February 2023—12 months into the project. As agreed, this working group should be an important element of a national initiative focused on emphasising horizontal approaches to strengthening the health system by moving away from assessments of vertical programmes (*démarche de qualité intégré*). Additional resource was allocated to this working group from the programme budget, and the team involved at the DPS level was expanded. This working group had representation from the partnership team and from relevant offices in the DPS (including INFOSAN, *appui technique* and *qualité des soins*). They provided more senior, strategic leadership for the work, led on regular site visits and were key in ensuring that the data workstream was well integrated into existing ministry and division work.

### Training

A bespoke training programme was developed in earlier work based on the WHO safe surgery 10 pillars, coupled with quality improvement methodology and contextualised for the Kongo Central. This 2-day course (safe surgery and quality improvement methodology; *Chirurgie en Sécurité et Amélioration de la Qualité des Soins (CESMAQS*)) was multidisciplinary, with surgeons, theatre nurses and anaesthetic care providers learning together. A train-the-trainers model was used with trainers from the four referral hospitals training clinicians at six district hospitals. The training included a module on surgical data and indicators as well as practical sessions on designing a quality improvement project.

Follow-up training specifically on surgical data was delivered at each of the 10 sites, including staff who had not attended the CESMAQS training. This training included nurses responsible for data entry and hospital managers responsible for data compilation before sending data to provincial headquarters. The training covered the ‘*canevas supplémentaire’* and subsequently the new indicators.

### New indicators

Seven surgical indicators were introduced into the existing data collection system (table 3). These were based on the Utstein list but crucially included additional indicators monitoring the quality of surgical care. This adaptation process started at the first stakeholder workshop and was further developed with the expanded working group in early 2023. It was agreed that the indicators that would be collected in Kongo Central should be in line with international recommendations, feasible for the local context (particularly in terms of resource and expertise), provide information on quality of care (and not just activity) and have the potential to be incorporated within the SNIS and DHIS2 national platforms. Indicator 4 (type of surgery) was already collected and was deemed important for healthcare planning. Indicators 6 (perioperative infection rates) and 7 (use of the WHO checklist) were included as a measure of the quality of care. Theatre logbooks at each of the 10 healthcare facilities were modified to ensure that data on postoperative infection rates and the use of the WHO checklist were incorporated. These indicators are being collected beyond the lifetime of this programme, demonstrating sustainable change in data processes.

**Table 3:** Kongo Central surgical data: collected monthly from the target healthcare facilities (secondary and tertiary)

Indicator	Data collected	Indicator
1. Geographical access*	Health zone population Facility with capacity to deliver the ‘Bellwether procedures’ Facility catchment area (‘population attractive’)	Population within a 5 km radius of the health facility able to carry out the Bellwether procedure
2. Healthcare workers*	Number of surgical care providers Number of specialist surgeons Number of anaesthetic care providers (nurses and technicians) Number of specialist anaesthetists Number of obstetric care providers Number of specialist obstetricians	Number of providers per 100 000 population
3. Surgical volume	Number of major cases Number of minor cases Total number of cases	Number of cases per 100 000 population
4. Type of surgery	Bellwether procedure: yes or no Type of surgery: trauma, obstetric and general surgery Type of surgery: urgent or planned	Number of cases in each category per 100 000
5. Perioperative mortality	Death <48 hours after the surgical intervention Death >48 hours after the intervention Death within 30 days of the intervention	Mortality (all causes) as a percentage of all cases
6. Perioperative infection rates	Number of surgical site infections	SSI (all types) as a percentage of all cases
7. Use of the WHO checklist	Percentage of cases using the checklist WHOBARS assessment of selected cases	WHO checklist use as a percentage of all cases

* Collected at the first site visit but not repeated on a monthly basis.

WHOBARS, World Health Organization Behaviorally Anchored Rating Scale.

### Quarterly site visits

Quarterly site visits, with programme staff and representatives from the DPS INFOSAN and quality of care offices, helped troubleshoot any problems, ensured data quality and completeness and provided additional training where necessary. Data were collected from all three data sources (theatre logbooks, SNIS and DHIS2), and the data were triangulated. There were regular discussions about the data between provincial leadership and clinicians during these visits.

### Traction at the national level

At a surgical roundtable event facilitated by the UK Foreign and Commonwealth and Development Office team in Kinshasa, national and provincial leaders were brought together to share learnings around the implementation of the safe surgery programme and, more specifically, the surgical data work. Surgical indicators, particularly in relation to quality of care, need to be included in national policies and processes. It was agreed that future work will ensure that surgical indicators are included in the next SNIS revision and in the national quality of care strategy (Plan National d’Amélioration de la Qualité de Soins (PNAQS)).

### Second senior stakeholder workshop

A second stakeholder meeting was held in Matadi in February 2024. An overview of the efforts to strengthen surgical data collection, analysis, interpretation and dissemination was presented by the DPS team and critically analysed. Those present at the Kinshasa roundtable meeting reported on the progress at the national level and outlined the national leadership support for work being done at the provincial level. Enthusiasm for continued data work, beyond the funding period, was expressed, and a further quarterly visit took place in June 2024. The senior stakeholder meetings highlighted that both national and facility level data are important to track system-wide metrics and patient-level metrics. With both frontline clinicians and provincial leaders present at both stakeholder meetings, this coordination has been strengthened.

### Lessons learnt

This initiative to focus on data and metrics, in the context of health systems strengthening through improving surgical care, was born out of a longstanding collaboration and strong relationships. The mutual trust between partners allowed for open discussions about the approach and to change practice when a better approach was identified. An example of this was the *‘canevas supplémentaire’*. Initially, it was thought as the best way to collect data related to surgical care that were not present in the existing data collection tools. Following its pilot and further review of the key indicators, this approach was modified.

Bringing the right people together for this work was vital. Early on, it was important to bring together frontline clinicians and healthcare leaders to guide programme design. However, it was only as the work progressed that it became clear exactly which offices within the division of health needed to be involved and that possible synergies became evident.

If this work was to be started again, we would place more emphasis on the integration with the nationally integrated quality approach (*démarche de qualité intégré*) and the national quality of care strategy (PNAQS). Alignment with these two programmes later on in the work allowed for a significant gear change in the approach and made the potential effect much clearer. Explainer table 4 summarises key reflections and lessons learnt through this programme of work.

**Table 4:** Key lessons learnt

Good clinical records and unique patient identifiers are key to good data systems.	The lack of good patient records and standardised identification numbers makes record keeping and subsequent analysis very difficult.
All levels of the health system need to engage with data work.	Frontline clinicians, as well as policy makers, want better clinical and system-level data to provide the best care for their patients. Health systems strengthening programmes should ensure that supporting good quality data is integrated into all programmes of work. A diverse group of stakeholders (including nurses, doctors, data managers and strategic leaders) need to engage in data collection, analysis, interpretation and dissemination.
Data need to be used to be meaningful.	Data need to be fed back to clinical staff and be used to improve care quality in real-time; creating a discussion between clinicians and policy makers catalyses improvement in data management.
It is vital to work with existing systems.	Our programme was embedded in the current practice and implemented small changes that could be introduced within the existing systems. We avoided the temptation to set up parallel data processes and were thus able to improve ownership and sustainability of data processes.

As we reflect on this work, strong relationships and iterative building of interventions have been valuable, and we would encourage others to adopt this approach where possible. This programmatic piece of work brought together key stakeholders to implement change guided by international recommendations on what data *should be* collected but with little guidance on *how to* do this. Experience in the Pacific Region was reported in 2017^10^ and in Colombia in 2020.^9^ In the Pacific region, the focus was on collecting the original LCoGS indicators, from existing healthcare data sets. The paper stresses the importance of collaboration, with Royal Colleges and Ministry among others, but does not go into detail about how this collaboration should come about. The work in Colombia reports on the LCoGS that does not seem to be part of a wider programme of continued data collection and analysis.

A pre-print article by Perez-Iglesias *et al*^11^ concluded that reporting rates for surgical indicators in the World Bank World Development Indicators are low, with surgical workforce density reporting at 0%. It concluded that data collection and reporting is vital but does not provide examples of implementation or recommendations about how this might be done. We have described our collaborative approach and the lessons learnt from this process.

### Discussion: Data are needed for health systems strengthening

Key elements of a health system strengthening intervention include the scope, scale, sustainability and effect of the intervention.^12^ This work to strengthen surgical data, at system-wide and individual patient levels, embraces these four critical elements. Firstly, the approach has been broad in scope in working across multiple WHO building blocks including workforce, health information, service delivery and leadership. It has also been disease agnostic and focused on a wide set of surgical conditions. Secondly, we have intervened at scale, working across multiple levels of the health system, including secondary and tertiary care levels and at the central and provincial ministry levels. This ministry engagement has resulted in provincial and national reach. Thirdly, we have taken a long-term approach, engaging strategically over many years, and always tackling systemic blockages to maximise the sustainable impact. Lastly, we have linked the surgical data work to effects—especially to the impact on surgical outcomes.

Improving surgical data collection, analysis, dissemination and subsequent data improvement interventional design and implementation, has been the fruit of sustained engagement from a wide spectrum of stakeholders, and we think that our work can be framed in the light of a learning health system with a vision to engage policy makers, researchers, service providers and patients in learning that uses and strengthens routinely collected data to conduct pragmatic, contextually appropriate research, promote rapid adoption of findings to improve quality and outcomes and promote continuous learning.^13^ The challenge we now face is to ensure that the collected data directly inform healthcare delivery and cycles of continued improvement.

The LCoGS called for the systematic collection of six surgical indicators relating to surgical preparedness, surgical delivery and financial effect.^6^ However, the subsequent collection of these indicators has been challenging, partly because little surgical data are routinely collected by Ministries of Health and partly due to the confused definition of some of the indicators^7^—an issue that was addressed in a recent indicator revision.^8^ This programme in the DRC has tackled some of the surgical data implementation challenges. Consistent engagement of stakeholders, including both national and provincial level ministry colleagues, has facilitated important discussions about what and how surgical data should be collected. Engaging front-line healthcare workers, including nurses completing theatre logs and departmental heads focused on quality improvement, has resulted in improvements in the quality of individual-level data and greater consistency of engagement in the process.

## Conclusion

Despite the recognised importance of good surgical data to effect change and influence policy, few publications have focused on efforts to strengthen surgical data collection, analysis, interpretation and dissemination. We present a programme in the DRC that has sought to strengthen the health system through improved data processes and encourage more shared learning to help guide practice. Our work has highlighted the importance of a strong partnership to facilitate taking a systems-level approach to improving surgical data.

## Supplementary material

10.1136/bmjgh-2024-017759 online supplemental file 1

10.1136/bmjgh-2024-017759 online supplemental file 2

10.1136/bmjgh-2024-017759 online supplemental file 3

10.1136/bmjgh-2024-017759 online supplemental file 4

10.1136/bmjgh-2024-017759 online supplemental file 5

10.1136/bmjgh-2024-017759 online supplemental file 6

## Data Availability

Data are available upon reasonable request.
