# Supplementary material for: Health systems strengthening in the Democratic Republic of Congo: the importance of surgical data
Source: BMJ Glob Health. 2025 Sep 4;10(9):e017759. doi: 10.1136/bmjgh-2024-017759 (PMC12414223; doi:10.1136/bmjgh-2024-017759)
Supplement: online supplemental file 1 [file bmjgh-10-9-s001.pdf]

WHO Safe surgery Saves Lives (2009)	Hospitals and public health systems will establish routine surveillance of surgical capacity, volume and results. WHO Member States should collect the following information:<br>1. The number of operating rooms in each country<br>2. The number of operations performed in operating rooms in each country<br>3. The number of trained surgeons and the number of trained anaesthesia professionals in each country<br>4. The number of deaths on the day of surgery<br>5. The number of in-hospital deaths following surgery
LCoGS Surgical Indicators (2016)	The Lancet Commission on Global Surgery recommends the following indicators for routine surveillance:<br>1. Proportion of the population that can access, within 2 hours, a facility that can do caesarean delivery, laparotomy and treatment of open long bone fracture (the Bellwether procedures)<br>2. The number of specialist surgical, obstetric and anaesthetic physicians who are working per 100,000 population<br>3. The number of procedures done in an operating theatre per 100,000 population per year<br>4. All cause death rate before discharge in patients who have undergone a procedure in an operating theatre using any form of anaesthesia divided by the total number of procedures, presented as a percentage, per year<br>5. Proportion of households protected against impoverishment from direct out-of-pocket payments for surgical and anaesthesia care<br>6. Proportion of households protected against catastrophic expenditure from direct out-of-pocket payments for surgical and anaesthesia care
Utstein Surgical Indicators (2021)	The Utstein consensus meeting refined the LCoGS indicators to recommend the following five indicators:<br>1. Proportion of a country's population with geographic access (within 2 hours) to a facility capable of providing surgical and anaesthesia care for the Bellwether procedures (caesarean delivery, laparotomy and surgical management of open long bone fracture)<br>2. Number of each of each of surgery, obstetric or anaesthesia providers who are actively practicing, per 100,000 population<br>3. The number of procedures done in an operating theatre, using any anaesthesia, per 100,000 population per year<br>4. Deaths from all causes before discharge (up to 30 days) in all patients who have received any anaesthesia for a procedure in an operating theatre, divided by the total number of procedures, per year, expressed as a percentage<br>5. Percentage of the population at risk of catastrophic expenditure if they were to require a surgical procedure

Explainer box : Existing frameworks for surgical metrics
