# Supplementary material for: Health systems strengthening in the Democratic Republic of Congo: the importance of surgical data
Source: BMJ Glob Health. 2025 Sep 4;10(9):e017759. doi: 10.1136/bmjgh-2024-017759 (PMC12414223; doi:10.1136/bmjgh-2024-017759)
Supplement: online supplemental file 2 [file bmjgh-10-9-s002.pdf]

King's Global Health Partnerships
in Democratic Republic of Congo

DECEMBER 2022

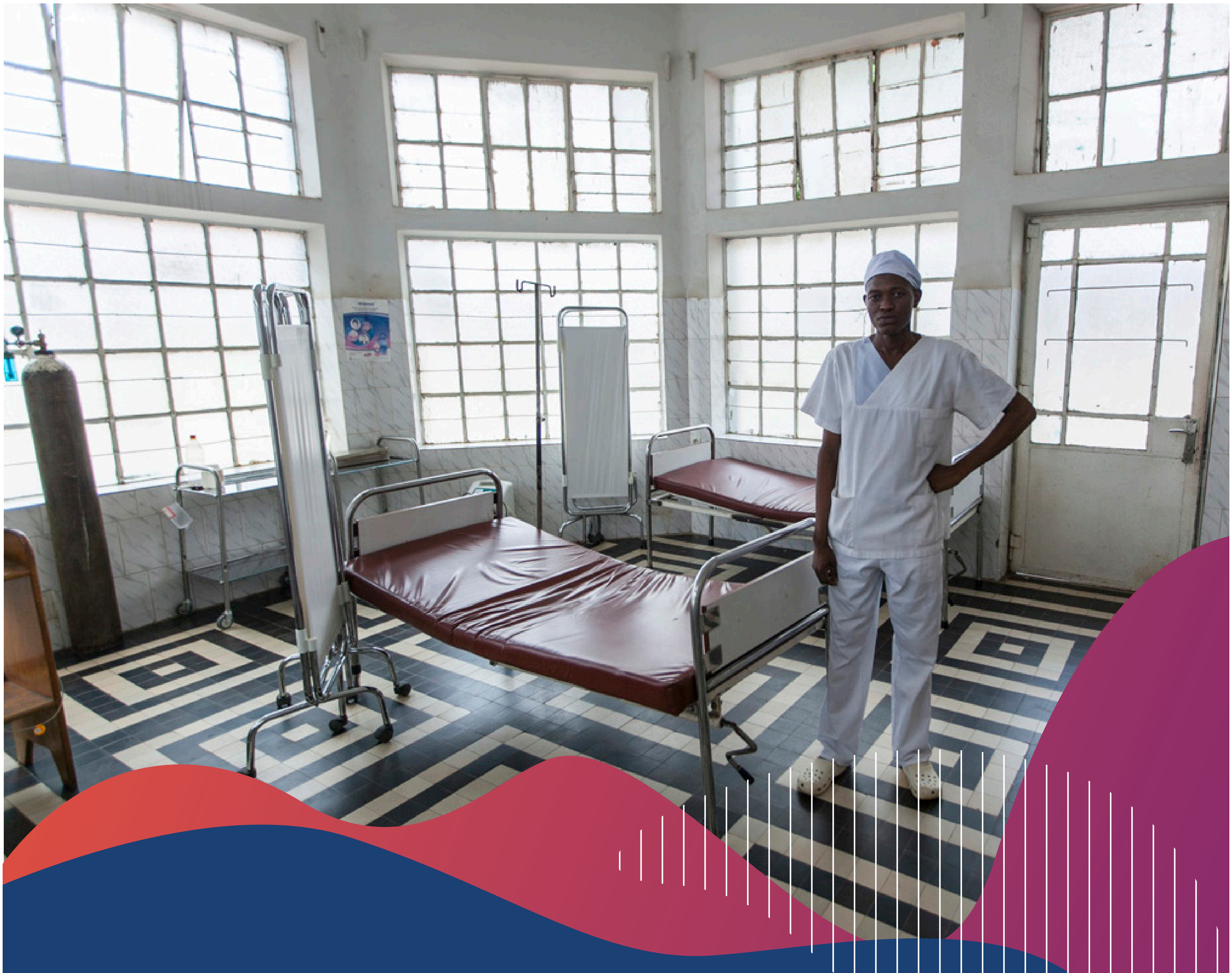

BASELINE STUDY SUMMARY

Surgical care in Kongo Central: Challenges and opportunities

KING'S HEALTH PARTNERS

KING'S College LONDON

The global need for surgery

The 2015 Lancet Commission on Global Surgery[1] estimated that five billion people lack access to safe, timely and affordable surgical and anaesthesia care. Surgery is a treatment which is needed across the entire range of human disease. It is needed when complications arise in pregnancy that require emergency obstetric care, to cure and control cancer, to treat physical injuries, as part of treatment of infectious diseases, and to treat numerous non-communicable diseases, such as cardiovascular disease, musculoskeletal and neurological disorders. Despite overwhelming evidence for the need for improvements to surgery, investment globally pales in comparison to the amount invested in other global health issues, such as malaria, tuberculosis and HIV.

Surgical care in Kongo Central

Kongo Central is a stable but neglected province in south-west Democratic Republic of Congo with an estimated population of six million. The province has a particularly high burden of road traffic collisions from the main road that connects the port towns to the capital, Kinshasa. Most of the population of Kongo Central lack access to high quality surgery, so case-fatality rates are high for common and easily treatable conditions such as fractures, appendicitis, hernia, obstructed labour, congenital anomalies, and breast and cervical cancer.

As part of a project to strengthen the safety of surgical care in the province, King's Global Health Partnerships and the Division Provincial de la Santé, conducted a baseline study of surgical capacity in eight hospitals: HGR Boma, HPR Kinkanda, IME Kimpese, St Luc Kisantu, CSR Kalamu, CSR Kiamvu, CSR Luila and CSR Kimpese. This report summarises the key findings.

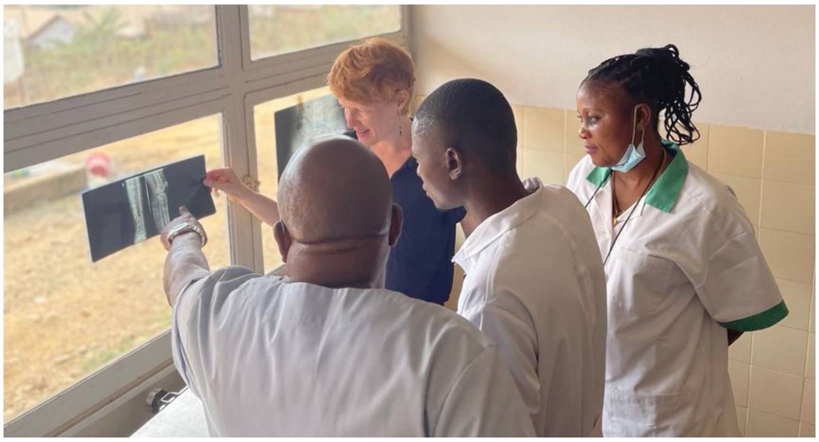

1 Global Surgery 2030: evidence and solutions for achieving health, welfare, and economic development (www.lancetglobalsurgery.org/_files/ugd/346076_ee70c0ea4fe54f3ca2b02dcc73c19afe.pdf)
2 Surgical care (www.who.int/teams/integrated-health-services/clinical-services-and-systems/surgical-care)

THE NEED FOR SURGERY IN NUMBERS

5 billion people
lack access to safe, timely and affordable surgical and anaesthesia care.[1]

9 out of 10 people
in low- and middle-income countries cannot access basic surgical care.[2]

6% of surgical procedures
take place in the poorest countries, where over a third of the world's population lives.[1]

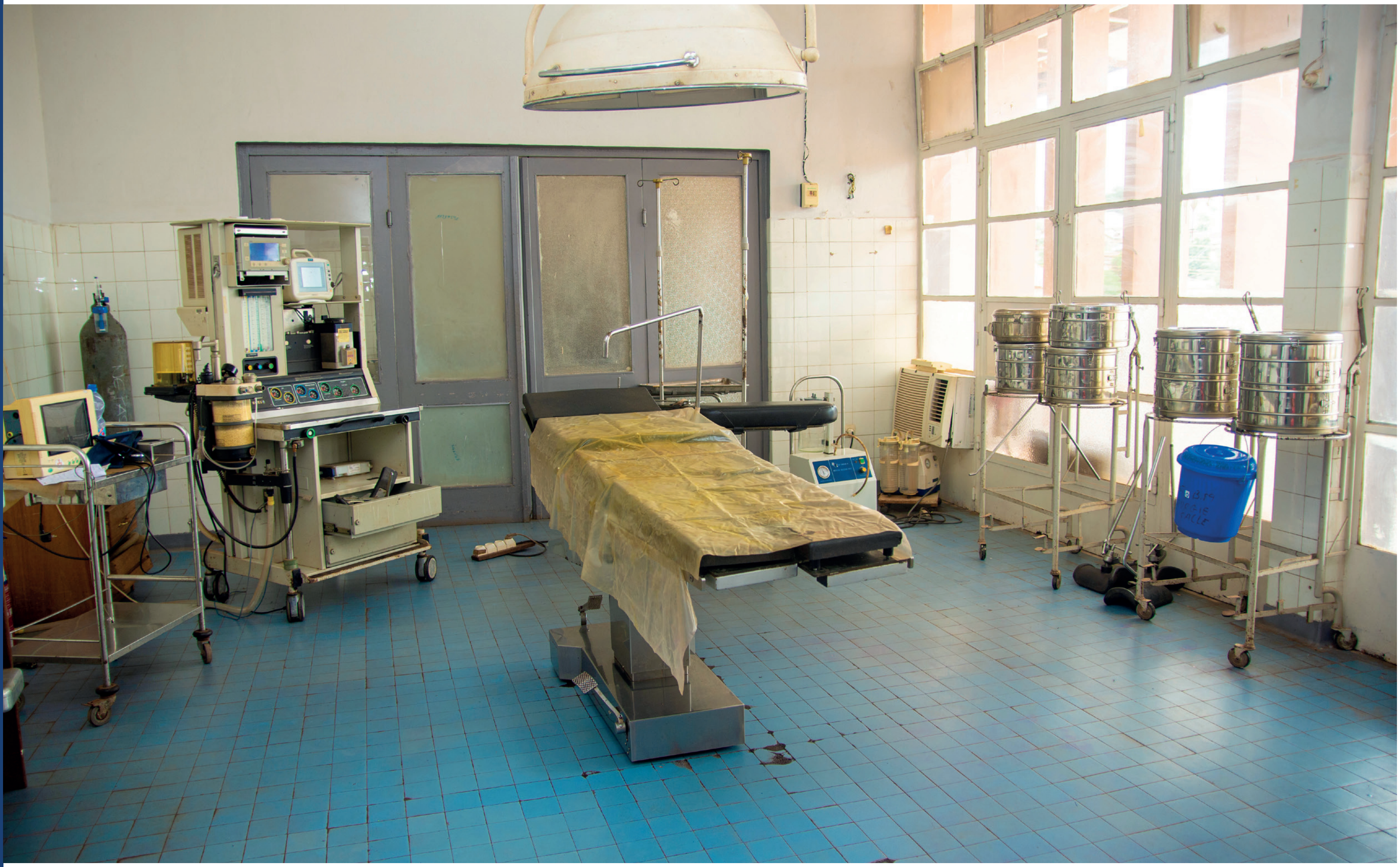

Finding 1: There is a large unmet need for surgical care in Kongo Central

The Lancet Commission on Global Surgery estimated that the minimum number of surgical procedures needed to address disease prevalence is 5,000 surgeries per 100,000 population, per year by 2030.

In Kongo Central, the hospitals surveyed conduct an average of 391 surgeries per 100,000 population, per year. The low number of surgeries shows there is a large unmet surgical need in Kongo Central – likely to be a result of high levels of poverty and lack of financial risk protection for patients, but also a lack of surgical capacity.

Surgical volume

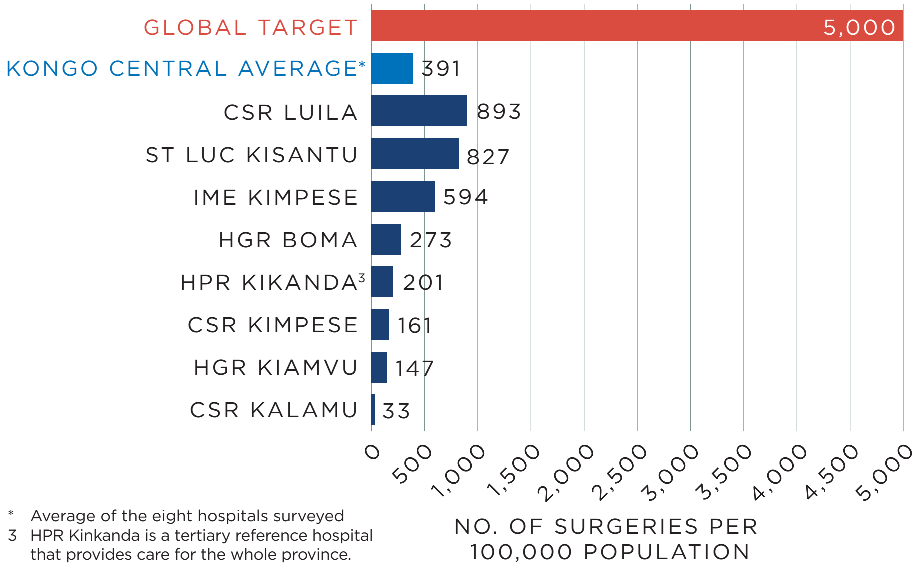

* Average of the eight hospitals surveyed

3 HPR Kinkanda is a tertiary reference hospital that provides care for the whole province.

Finding 2: The main types of surgery conducted are caesarean section and trauma surgery

Types of surgery conducted in eight hospitals surveyed

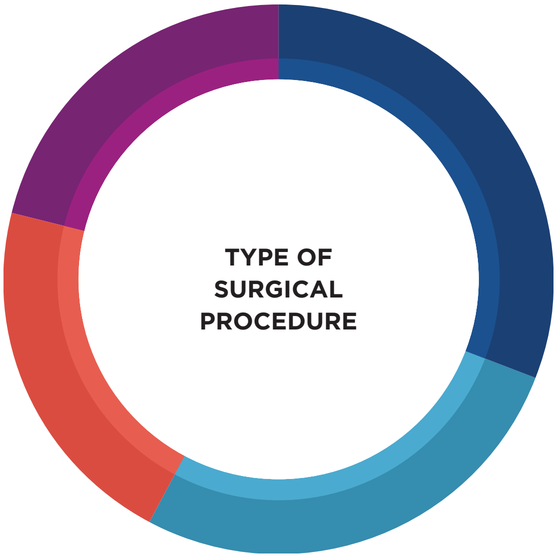

TRAUMA SURGERY 31%
Includes all interventions to treat acute injuries (such as fracture, intra-abdominal bleed) and includes all interventions to treat the sequelae of injury (e.g. osteomyelitis from open fracture); includes all specialties (musculoskeletal (MSK)/ orthopaedic, plastics, general surgery, urology, maxillofacial; there is no neurosurgical trauma care).

CAESAREAN SECTION 27%
Includes planned and emergency c-sections, and surgery to manage obstetric emergencies (placenta previa, post-partum haemorrhage and uterine rupture).

GENERAL SURGERY 21%
All general surgery cases, including laparatomies, hernia repair, abscess incision and drainage (I&D).

OTHER 21%

The data demonstrates that the majority of surgical care is to treat acute and emergency conditions, related to trauma and injury as well as obstetric emergencies. Over a third (31%) of the surgery conducted in the hospitals we surveyed were related to trauma and 27% were caesarean sections. This shows that the majority of surgery conducted in the province is emergency, life-saving procedures.

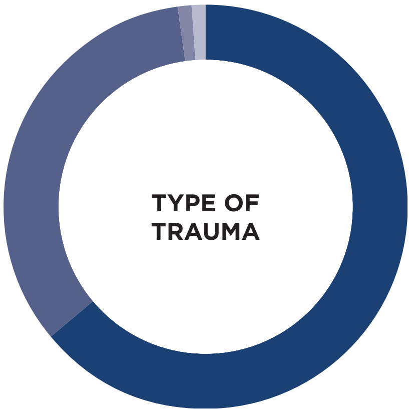

TRAUMA MUSCULOSKELETAL (MSK)/ORTHOPAEDICS 64%
Fracture reduction, fracture fixation, treatment for osteomyelitis, removal of metalwork from previous fixation.

TRAUMA PLASTICS 34%
Wound debridement, split skin graft.

TRAUMA GENERAL SURGERY 1%
Laparotomy for trauma.

TRAUMA GENERAL 1%

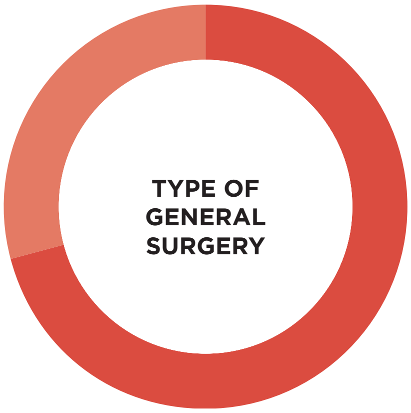

ACUTE 71%
Emergency laparotomies for perforated ulcer or obstruction, abscess, appendicectomy.

ELECTIVE 29%
Hernia repair.

Finding 3: There is a critical shortage of surgical specialists

The 2015 Lancet Commission on Global Surgery set a target for all countries to have at least 20 fully trained surgical, anaesthetic, and obstetric physicians per 100,000 of the population, by 2030.[1]

In the eight hospitals surveyed in Kongo Central there are 11 surgical specialists (six qualified surgeons, three qualified anaesthesiologists and two qualified obstetricians) for the 1,186,700 people that these facilities serve. Giving a specialist surgical workforce density of one provider per 100,000 of the population. The few post-graduate trained specialists the province does have are concentrated in Kongo Central's urban areas. The majority of surgical care is therefore delivered by non-specialist surgical providers – doctors with no postgraduate training, covering all surgical and obstetric emergencies, and anaesthetic nurses or technicians working without specialist support.

LANCET COMMISSION GLOBAL TARGET:

20 surgical specialists per 100,000 population

AVERAGE IN EIGHT TARGET FACILITIES:

1 surgical specialist per 100,000 population

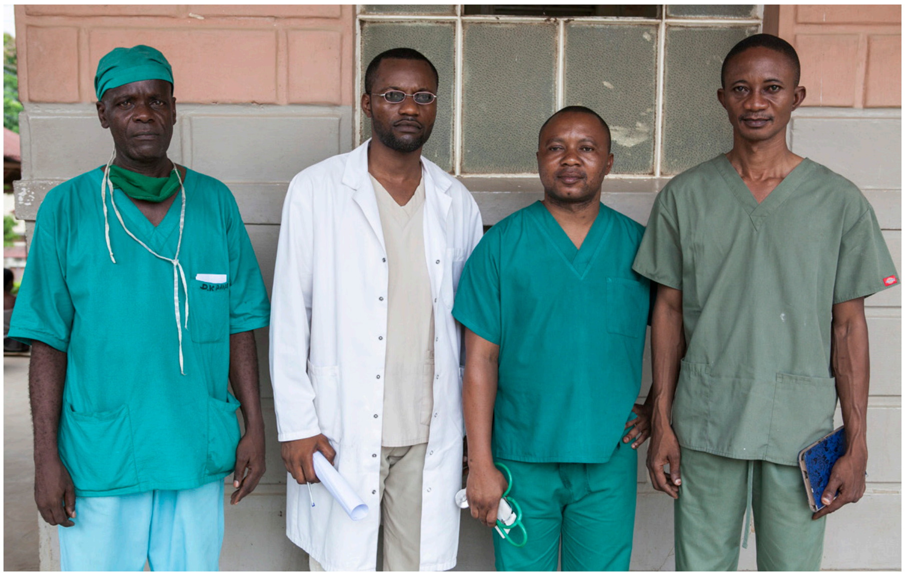

Specialist surgical workforce density per 100,000 population in eight hospitals in Kongo Central province, DRC; compared to the Lancet Commission global target

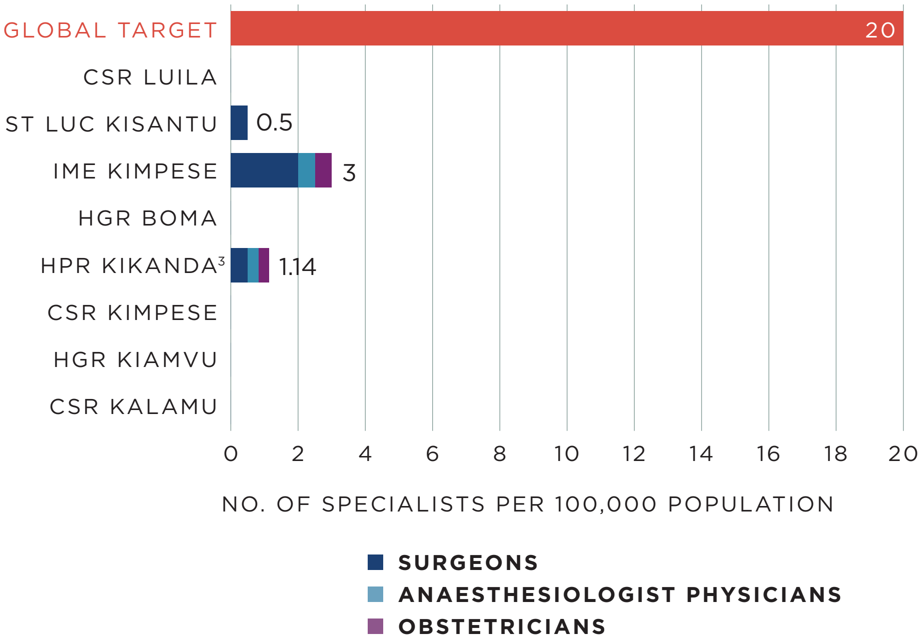

1 Global Surgery 2030: evidence and solutions for achieving health, welfare, and economic development (www.lancetglobalsurgery.org/_files/ugd/346076_ee70c0ea4fe54f3ca2b02dcc73c19afe.pdf)
3 HPR Kinkanda is a tertiary reference hospital that provides care for the whole province.

Finding 4: There is a critical shortage of infrastructure and equipment for surgery

None of the hospitals surveyed have all of the critical infrastructure and equipment needed to conduct safe surgery as defined in the WHO Tool to Assess Emergency and Essential Surgical Care.[4] Four of the eight facilities surveyed have no blood bank to support blood transfusions, six do not have a reliable source of water and five do not have a reliable source of electricity.

Availability of critical infrastructure and equipment needed to conduct safe surgery at each of the eight hospitals surveyed

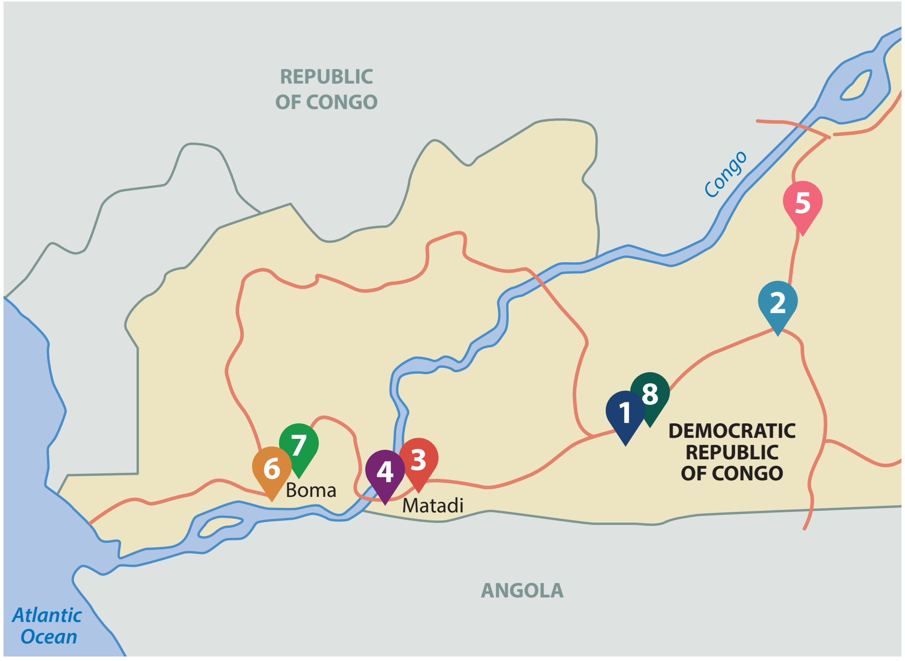

	Population served	Blood bank	No. of operating rooms	Oxygen	Running Water	Electricity	Anaesthesia machine	Dedicated area for emergency care
1 IME KIMPESE	199,233	Yes	5	Yes	Yes	Yes	Yes	Yes
2 ST LUC KISANTU	214,780	Yes	4	Yes	Not reliable	Not reliable	Yes	Yes
3 HPR KINKANDA[3]	312,372	Yes	3	Yes	Not reliable	Not reliable	No	Yes
4 CSR KIAMVU	147,056	No	1	Not reliable	Not reliable	Not reliable	No	No
5 CSR LUILA	11,646	No	0	None	None	None	No	No
6 HG BOMA	262,487	No	3	Not reliable	Not reliable	Not reliable	No	No
7 CSR KALAMU	24,276	Yes	1	No	Not reliable	None	No	No
8 CSE KIMPESE	14,871	No	1	No	Not reliable	Not reliable	No	No

3 HPR Kinkanda is a tertiary reference hospital that provides care for the whole province.
4 https://www.who.int/teams/integrated-health-services/patient-safety/research/safe-surgery

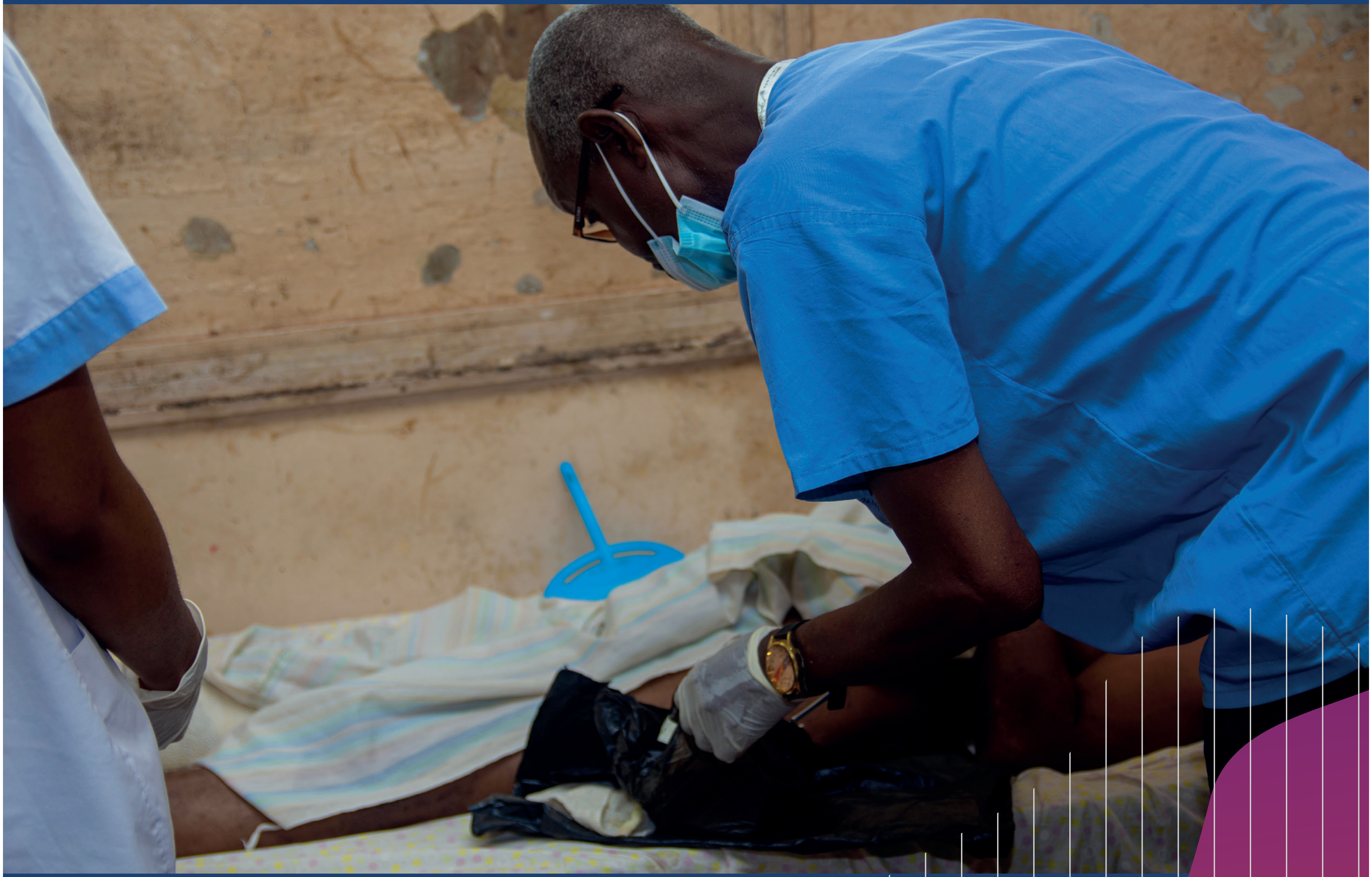

Safe Surgery Project

This research was conducted as part of the project: Developing safe-surgery practice in Kongo Central province in Democratic Republic of Congo, funded by the Else Kröner-Fresenius-Stiftung. Building on a seven-year partnership with the *Ministère Provincial de la Santé*, the project is taking a multi-level approach to strengthen surgical care. We are:

- building the capacity of the **individuals** delivering surgery, by providing supplementary training for the largely unspecialised workforce in infection prevention control, safe anaesthetic care and theatre practice
- strengthening the **institutions** delivering surgery, by providing low cost and sustainable surgical equipment, such as pulse oximeters and sterilisers
- supporting **health system** improvements by strengthening hospital and government collection and use of data on surgery.

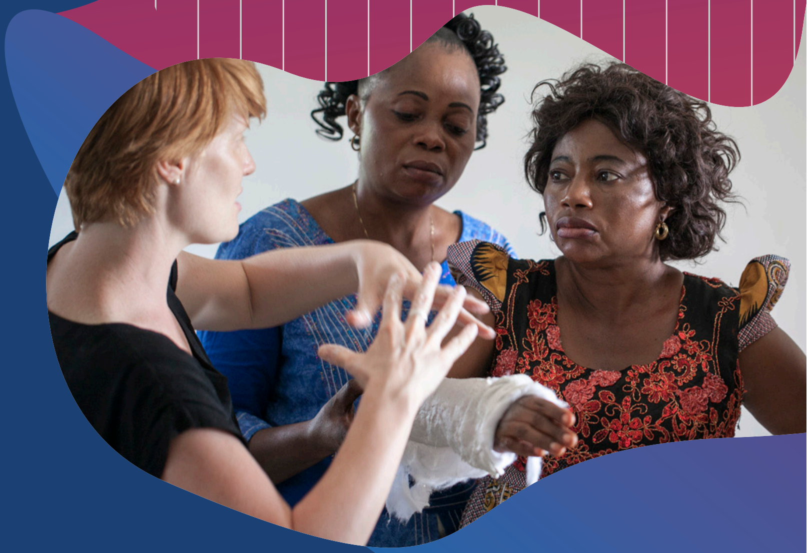

King's Global Health Partnerships

King's Global Health Partnerships works with health facilities, academic institutions and governments to strengthen health systems and improve the quality of care in four countries: Somaliland, Sierra Leone, the Democratic Republic of Congo and Zambia. We bring together health, academic and international development expertise from King's College London, the UK's National Health Service (NHS) and our international partners to: Educate, train and support healthcare workers, strengthen healthcare and training institutions, and enhance national health policies and systems.

The data presented was collected in April 2022 by King's Global Health Partnerships in collaboration with the *Division Provincial de la Santé*. It reflects surgical activity in eight hospitals which cover a broad geographic area and include the four main referral hospitals but do not cover all 31 health zones in the province. There may be more than one facility providing surgical care for a particular health zone and, particularly in urban areas, there may be overlap.

THANKS AND ACKNOWLEDGEMENTS

With thanks to the Else Kröner-Fresenius-Stiftung for their generous support to this project. Thank you to the *Ministère Provincial de la Santé* and the *Division Provincial de la Santé*. Thank you also to all those who collected and analysed the data included in this report.

KING'S COLLEGE LONDON

King's College London
Centre for Global Health and Health Partnerships
5th Floor Addison House
Guy's Campus
London SE1 1UL

T: 020 7848 5060
E: kghp@kcl.ac.uk
W: kcl.ac.uk/KGHP
@KingsGHP | @KingsKCP

Else Kröner Fresenius Stiftung

MINISTERE DE LA santé
