# Supplementary material for: Health systems strengthening in the Democratic Republic of Congo: the importance of surgical data
Source: BMJ Glob Health. 2025 Sep 4;10(9):e017759. doi: 10.1136/bmjgh-2024-017759 (PMC12414223; doi:10.1136/bmjgh-2024-017759)
Supplement: online supplemental file 3 [file bmjgh-10-9-s003.pdf]

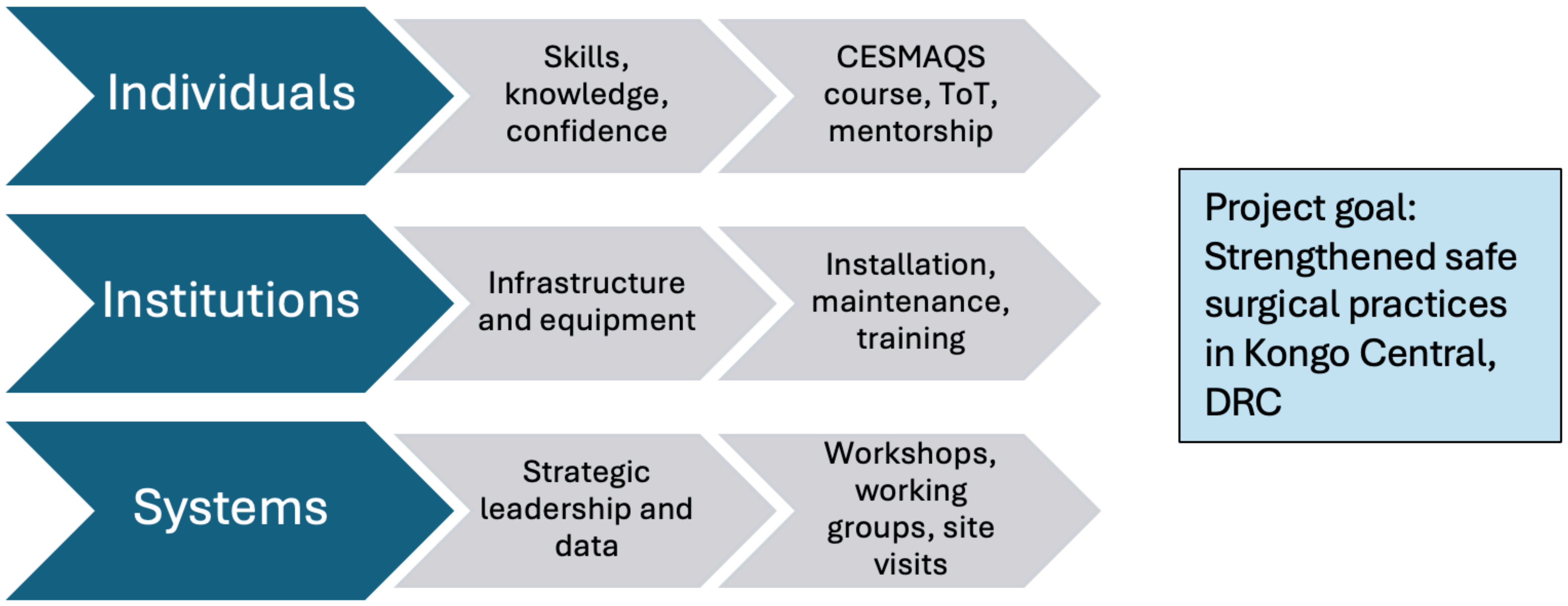

Figure 2: Illustrated theory of change: Working at three levels to improve surgical care in Kongo Central, DRC
