# Supplementary figures and images for: Health systems strengthening in the Democratic Republic of Congo: the importance of surgical data
Source: BMJ Glob Health. 2025 Sep 4;10(9):e017759. doi: 10.1136/bmjgh-2024-017759 (PMC12414223; doi:10.1136/bmjgh-2024-017759)

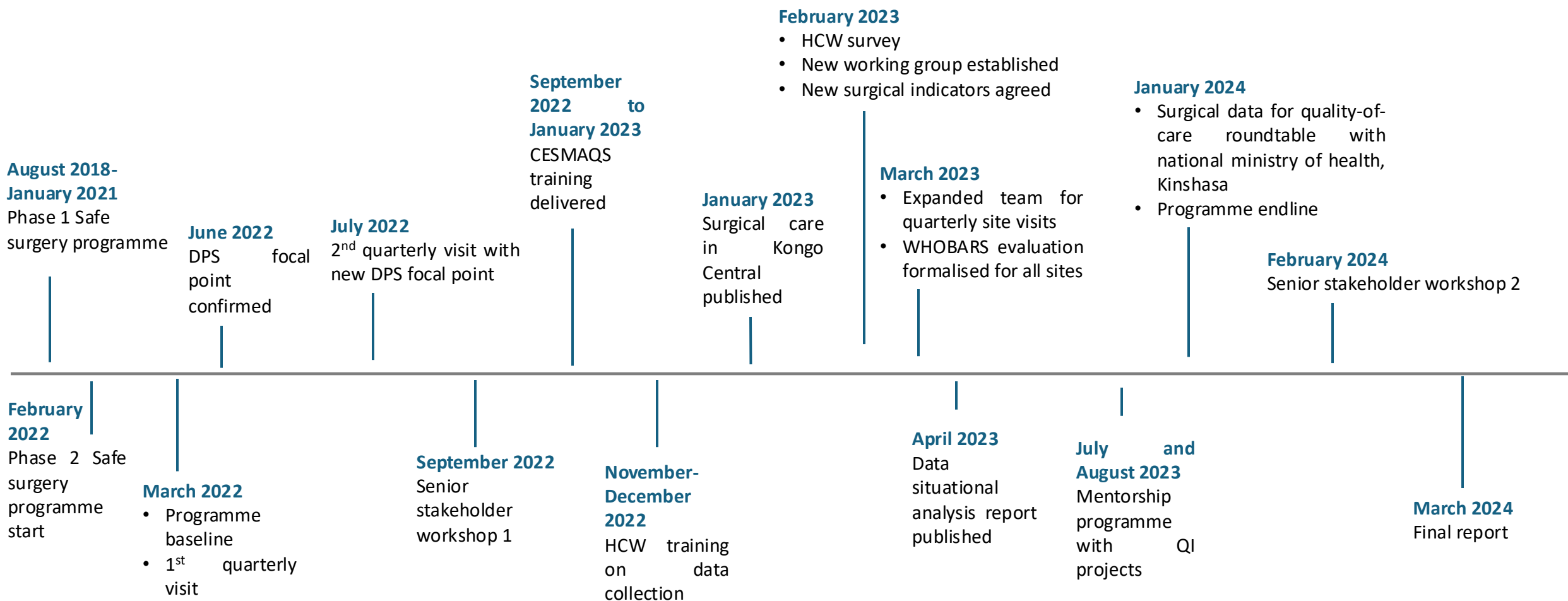

Supplement: online supplemental file 4 [file bmjgh-10-9-s004.pdf]
