# Supplementary material for: Health systems strengthening in the Democratic Republic of Congo: the importance of surgical data
Source: BMJ Glob Health. 2025 Sep 4;10(9):e017759. doi: 10.1136/bmjgh-2024-017759 (PMC12414223; doi:10.1136/bmjgh-2024-017759)
Supplement: online supplemental file 5 [file bmjgh-10-9-s005.pdf]

King's Global Health Partnerships

MINISTERE DE LA santé

Surgical, obstetric, trauma and anaesthetic data collection and management in Kongo Central DRC

Situational analysis

ELIZABETH TISSINGH • ACHIM MAMBU VANGU • JOENEL MFUNDU MBUANGI • MICHEL NLANDU • GUYLAIN

KING'S HEALTH PARTNERS

KING'S College LONDON

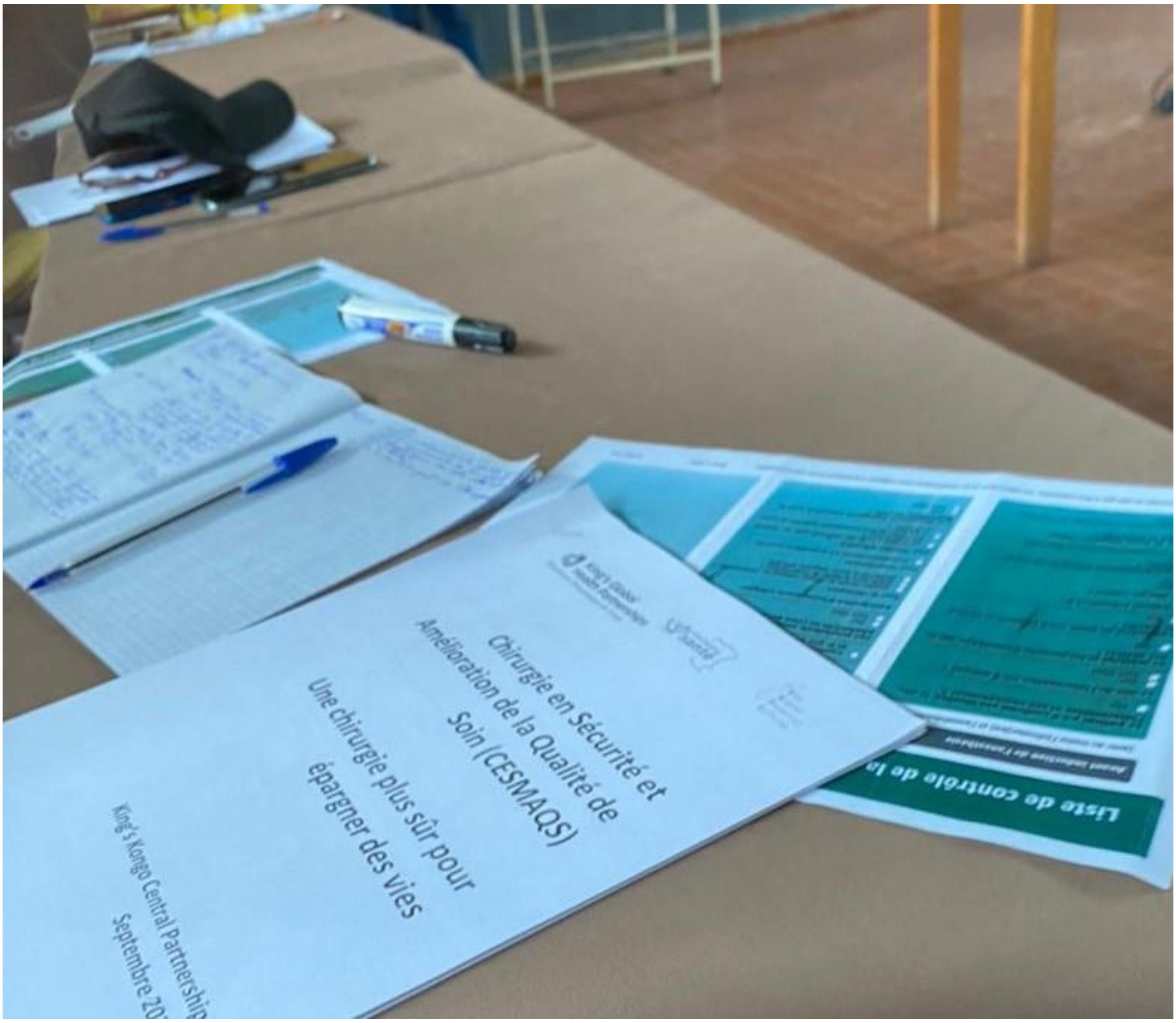

CESMAQS training at CSR Luila in November 2022

ABBREVIATIONS

- CESMAQS — Chirurgie en sécurité et amélioration de la qualité de soin
- DHIS2 — District Health Information Software 2
- DPS — Division Provincial de la Santé (Provincial Division of Health)
- DRC — Democratic Republic of the Congo
- FOSA — Formation sanitaire
- HCW — Health Care Worker
- KGHP — King's Global Health Partnerships
- KKCP — Kings Kongo Central Partnership
- MdS — Ministère Provincial de la Santé (Provincial Ministry of Health)
- MCZ — Médecin chef de zone
- MEL — Monitoring Evaluation Learning
- SNIS — Système National d'Information Sanitaire (National Health Information System)
- SOTA — Surgical, obstetric, trauma, anaesthetic
- UHC — Universal Health Coverate
- WHO — World Health Organisation

King's Global Health Partnerships

Contents

Introduction 4
Methodology 6
1. Baseline interviews and Healthcare Facility visits 6
2. Surgical data workshop 6
3. Healthcare worker survey 6
Surgical data prioritisation 7
Summary of findings 14
Recommendations 15

Introduction

SAFE SURGERY

Surgical, obstetric, trauma and anaesthetic (SOTA) care is an essential component of Universal Health Coverage (UHC)[1]. The WHO Safe Surgery Saves Lives initiative[2] seeks to improve the safety of surgical care around the world by defining a core set of safety standards outlined in ten pillars, including patient monitoring with oxygen saturation probes; infection, prevention and control; and implementation of the WHO Safe surgery checklist. A key recommendation is the collection of surgical data to monitor patient safety and for use in policy development.

SURGICAL METRICS IN THE GLOBAL SURGERY AGENDA

Global health has traditionally focused on indicators for HIV, TB and malaria, and maternal and child health, with data collection and management priorities often being donor led. Although SOTA care is an integral part of UHC, surgical metrics are often not well understood and poorly utilised.

There has been an increased recognition of the need for data in advancing the global surgery agenda[3]. The initial work by the WHO Safe Surgery Saves Lives initiative[2] was further developed by the Lancet Global Surgery Commission 2015 which outlined 6 indicators[4 5]. These were further refined by the Utstein Consensus[6].

The Lancet Commission on Global Surgery[4] outlines four key principles for data collection: simplicity, wide applicability, relevance to public health, and unintended negative consequences of measurement reduced to a minimum. A more recent publication from work in Ethiopia asked the following question: “How do we choose global health indicators that coincide with community priorities and improve local systems?”[7]. They highlighted the following principles from a paper by Larson et al. on global health indicators[8]: definition, validity, feasibility, utility. There is limited information in the literature on how this data should be collected and analysed or how it fits into existing health information systems.

Objective 10 of the WHO Safe Surgery Saves Lives Initiative states that hospitals and public health systems will establish routine surveillance of surgical capacity, volume and results. The recommendation is that WHO Member States should collect the following information: the number of operating rooms in each country, the number of operations performed in operating rooms in each country, the number of trained surgeons and the number of trained anaesthesia professionals in each country, the number of deaths on the day of surgery, the number of in-hospital deaths following surgery[9].

[1] Manon Pigeolet,1,2,3 Selam Degu,1 Isabella Faria,1 Matthew T. Hey,1 Tayana Jean-Pierre,1 Don E. Lucerno-Prisno,1 Ali Jafarian,4 Natalia Kanem,5 John G. Meara,1 Lia Tadesse Gebremedhin,6 Cherian Varghese,7 Tarsicio Uribe-Leitz,1 and Kee B. Park1 Universal health coverage: a commitment to essential surgical, obstetric, and anesthesia care, World Health Summit 2021 (PD 20) BMC Proc. 2023; 17(Suppl 6): 4.

[2] World Health Organisation Safe surgery saves lives 2009 https://www.who.int/teams/integrated-health-services/patient-safety/research/safe-surgery

[3] John Rose, Harsha Malapati, Chao Long, Matchecane Cossa, Kavitha Ranganathan *Metrics without borders: advancing the global surgery agenda through data* Journal of Public Health and Emergency Vol 4 (December 2020)

[4] Insights publication: Global Surgery 2030: evidence and solutions for achieving health, welfare, and economic development (www.lancetglobalsurgery.org/_files/ugd/346076_ee70c0ea4fe54f3ca2b02dcc73c19afe.pdf)

[5] Evaluating the collection, comparability and findings of six global surgery indicators H. Holmer, A. Bekele, L. Hagander, E. M. Harrison, P. Kamali, J. S. Ng-Kamstra, M. A. Khan, L. Knowlton, A. J. M. Leather, I. H. Marks, J. G. Meara, M. G. Shrime, M. Smith, K. Søreide, T. G. Weiser, J. Davies First published: 20 December 2018 https://doi.org/10.1002/bjs.11061 https://bjssjournals.onlinelibrary.wiley.com/doi/10.1002/bjs.11061

[6] Davies JI, Gelb AW, Gore-Booth J, Martin J, Mellin-Olsen J, Åkerman C, et al. (2021) Global surgery, obstetric, and anaesthesia indicator definitions and reporting: An Utstein consensus report. PLoS Med 18(8): e1003749. https://doi.org/ 10.1371/journal.pmed.1003749 Published: August 20, 2021

[7] OCTOBER 31, 2022 BY KATHERINE IVERSON, MD AND KAYLEIGH R. COOK Who’s Counting Anyways? An Exploration of Metrics in Global Surgery Association of Academic Surgery https://www.aasurg.org/blog/whos-counting-anyways-an-exploration-of-metrics-in-global-surgery/

[8] Larson, C. (2004). Global health indicators: An overview. *Canadian Medical Association Journal, 171*(10), 1199-1200. doi:10.1503/cmaj.1021409 Lancet 2015; 386: 569–624 Published Online April 27, 2015 http://dx.doi.org/10.1016/ S0140-6736(15)60160-X

[9] World Health Organisation Safe surgery saves lives 2009 https://www.who.int/teams/integrated-health-services/patient-safety/research/safe-surgery

The Lancet Commission on Global Surgery highlights the following six surgical indicators: geographic accessibility of surgical facilities, density of specialist surgical providers (surgeons, anaesthetists, and obstetricians), number of surgical procedures provided per 100,000 population, perioperative mortality rates, risk of impoverishing expenditure when surgery is required, and risk of catastrophic expenditure when surgery is required[10]. The Utstein consensus[11] refined the Lancet surgical indicators and reduced them to five: volume, access, workforce, perioperative mortality, and catastrophic expenditure.

THE SAFE SURGERY SAVES LIVES PROGRAMME IN KONGO CENTRAL

King's Global Health Partnerships has worked with the Kongo Central Provincial Ministry of Health and Division of Health to improve the quality of surgical, obstetric, trauma and anaesthetic care in the Kongo Central province since 2018. The current Safe Surgery programme is funded by the EKFS Foundation and runs from March 2022 to 2024. As part of this programme, an evaluation has been carried out to understand the current health data collection and processing system, and improve its ability to measure surgical indicators in order to improve the quality of care.

KONGO CENTRAL PROVINCE

The Kongo Central province is in the southwest of the DRC, serving a population of close to six million. It is divided into 31 health zones. Central healthcare governance is with the Provincial Ministry of Health (Ministère Provincial de la Santé, MdS) and the division of health (Division Provincial de la Santé, DPS), both based in Matadi.

An initial eight healthcare facilities were analysed as part of this project. This was later increased to ten. A summary of these health facilities is found in Table 1.

Healthcare facility	Location	Urban or rural	Type of facility	Started safe surgery work*	Paired with	Population facility serves
1. HPR Kinkanda	Matadi	Urban	Tertiary referral hospital Government	August 2018	HG Kiamvu	312,372
2. HG Kiamvu	Matadi	Urban	District level hospital	January 2023	HPR Kinkanda	147,056
3. HG Boma	Boma	Urban	Referral hospital Government	August 2018	CSR Kalamu	262,487
4. CSR Kalamu	Boma	Urban	District level hospital	September 2022	HG Boma	24,276
5. IME Kimpese	Kimpese	Rural	Referral hospital Faith based	August 2018	CS Kimpese<br>CECO<br>CS Lamba	199,233
6. CS Kimpese	Kimpese	Rural	District level health centre Government	October 2022	IME Kimpese	14,871
7. CECO	Kimpese	Rural	District level health centre Government	October 2022	IME Kimpese	14,871
8. CS Lamba	Kimpese	Rural	District level health centre Government	October 2022	IME Kimpese	14,871
9. St Luc Kisantu	Kisantu	Urban	Referral hospital Faith based	August 2018	CSR Luila	214,780
10. CSR Luila	Luila	Rural	District level health centre Government	November 2022	St Luc Kisantu	11,646

*Time at which HCWs received CESMAQS training.

Table 1 Summary description of the ten healthcare facilities in the Safe Surgery programme

10 346076_713dd3f8bb594739810d84c1928ef61a.pdf (lancetglobalsurgery.org)

11 Davies JI, Gelb AW, Gore-Booth J, Martin J, Mellin-Olsen J, Åkerman C, et al. (2021) Global surgery, obstetric, and anaesthesia indicator definitions and reporting: An Utstein consensus report. PLoS Med 18(8): e1003749. 10https://doi.org/.1371/journal.pmed.1003749 Published: August 20, 2021

Methodology

A mixed methods approach has been used to understand and evaluate the flow of health care data in the province. This was done at healthcare facility level (ten secondary and tertiary healthcare facilities) and at central level in the capital of the province, Matadi. Work was carried out from March 2022 to April 2023. The three elements that were used for this situational analysis are summarised below:

1. BASELINE INTERVIEWS AND HEALTHCARE FACILITY VISITS

Visits were carried out in the initial eight healthcare facilities in March 2022. A further two healthcare facilities were added to the project at a later stage; they were not included in the initial assessment.

The healthcare facilities were visited and evaluated by a team, including the King's Kongo Central Partnership Lead, the KKCP In-Country Coordinator, the Project Data Manager and a representative from the Division of Health. Logbooks and patient records were reviewed and evaluated. In-depth interviews were carried out with doctors, nurses, hospital managers and health data officers.

Full findings from the baseline interviews during visits to healthcare facilities, formed part of the March 2022 report.

2. SURGICAL DATA WORKSHOP

A workshop bringing together senior stakeholders was held in Matadi in September 2022, facilitated by the KKCP team. This was attended by the medical directors of the health zones involved in the project, the focal points of the four referral healthcare facilities involved in the project (all surgeons), the focal point at the DPS for the project, the director of the bureau INFOSAN (health information office) at the DPS, the medical director of the DPS, the health advisor at the ministry of health, KGHP staff and volunteers.

The following key actions took place:

- Findings from the baseline site visit and interviews were presented
- A presentation was given on surgical indicators and new data collection points were agreed
- The data process map was presented and refined on the basis of feedback

The workshop revealed that there was a lack of knowledge of surgical metrics (including collection, analysis and use) but a strong desire to engage in the process of improving this in the province.

3. HEALTHCARE WORKER SURVEY

A survey was developed to gather information from clinicians, health information officers and senior leadership in the following domains:

- Familiarity with standard surgical indicators
- Current data management process
- Current data quality
- Motivation of the respondents to improve surgical data management processes

Surgical data prioritisation

It is clear that most healthcare data collection, evaluation and use is led by donors. Interviewees highlighted that data collected is done so either because it is part of the existing Système National d'Information Sanitaire (National Health Information System, SNIS) process or because donors have asked for specific information. Where data in the SNIS is incomplete for donor purposes, it is supplemented with an additional data collection tool (known as a 'canevas supplementaire'). This is the case for information on childhood immunisation for UNICEF and information on nutrition.

"We don't collect it [surgical data] because donors don't ask for it."

Kongo Central healthcare worker

The senior stakeholder workshop, in-depth interviews and data survey all conclude that Surgical, Obstetric, Trauma and Anaesthetic (SOTA) data is not prioritised within health care data management at provincial or national level. Many respondents said that this reflected an overall lack of strategic thinking around conditions outside communicable disease and maternal and child health. They also point towards the lack of priority given to overall healthcare and quality of care with a damaging focus on vertical programmes.

HUMAN RESOURCE

Multiple healthcare workers, with varying degrees of training, were involved at healthcare facility, health zone and provincial level. Some were health information specialists but many are frontline healthcare workers who are involved in data entry and analysis as part of their clinical roles. In most cases, it is the surgical and obstetric teams who are involved in data entry. Anaesthetic teams have limited involvement.

Table 2 provides a summary of the human resource involved at each level for SOTA data management.

Facility level	Clinicians	Data specialists	Healthcare data teams
**Primary facility level (CS* and CSR*)**	Theatre nurse in charge<br>Ward nurse in charge<br>Doctor<br>Surgeon	Nurses with additional training	
**Secondary facility level (HG*)**	Theatre nurse in charge<br>Ward nurse in charge<br>Doctor<br>Surgeon	Nurses with additional training<br>Data officers<br>Statisticians	Data committee<br>Statistics office
**Tertiary/ referral facility (HPR*)**	Theatre nurse in charge<br>Ward nurse in charge<br>Doctor<br>Surgeon	Nurses with additional training<br>Data officers<br>Statisticians	Data committee<br>Statistics office

* CS = centre de santé; CSR = centre de santé de reference; HG = hôpital general; HPR = hôpital provincial de reference

Table 2 Summary of people involved in health care data collection and analysis

Results from the survey (presented in Table 3) show that most respondents are not familiar with most standard surgical indicators. Respondents have some knowledge of the WHO core surgical dataset, which may be due to the workshop session on this dataset.

Standard Surgical Indicators	Respondents answering "little known" or "well-known"	Respondents answering "not at all familiar"
WHO's essential data set on surgery	60%	40%
The Lancet Commission on Surgical Indicators	10%	70%

Standard Surgical Indicators	Respondents answering "little known" or "well-known"	Respondents answering "not at all familiar"
Utstein consensus on Surgical Parameters	0%	50%
RDC Surgical Indicators Plan	10%	50%

Table 3 Table to show familiarity of respondents with Standard Surgical Indicators

It was also found that while 90% of the respondents have received training for health data management, only 20% respondents have received training in surgical data management. 50% respondents have completed their training more than 3 years ago. Whilst most respondents believe that there is a surgical data system in place in the DRC and know where to access surgical data in the healthcare dataset, not many people are fully aware of the process of data management and only 50% respondents believe that the system is efficient and fit-for-purpose as seen in Figure 1.

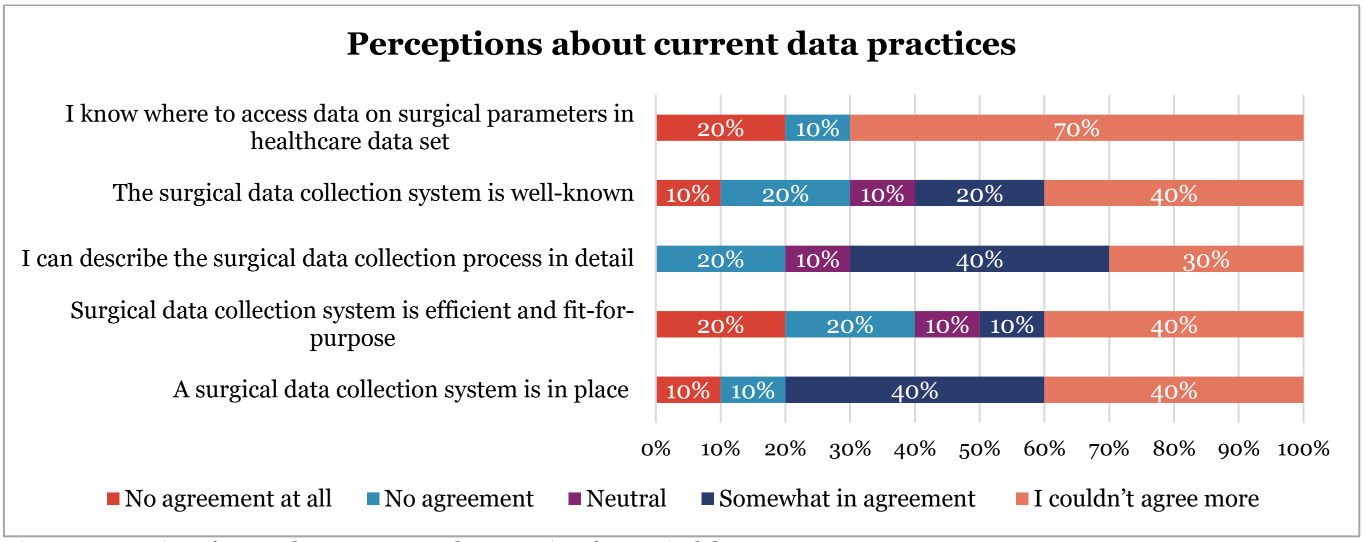

Figure 1 Perception of respondents on current data practices for surgical data

All respondents were highly motivated to learn more about surgical data management and believed that they could make significant changes in data management through better training (Table 4). 90% respondents believe that they can do something to improve current surgical data management in the DRC (Figure 2).

Statement	"No agreement at all" or "No Agreement"	"I couldn't agree more"
I'd like to know more about surgical data management	0%	100%
If I had better training I could make significant changes	0%	100%

Table 4 Motivation of respondents to improve data management for surgical data

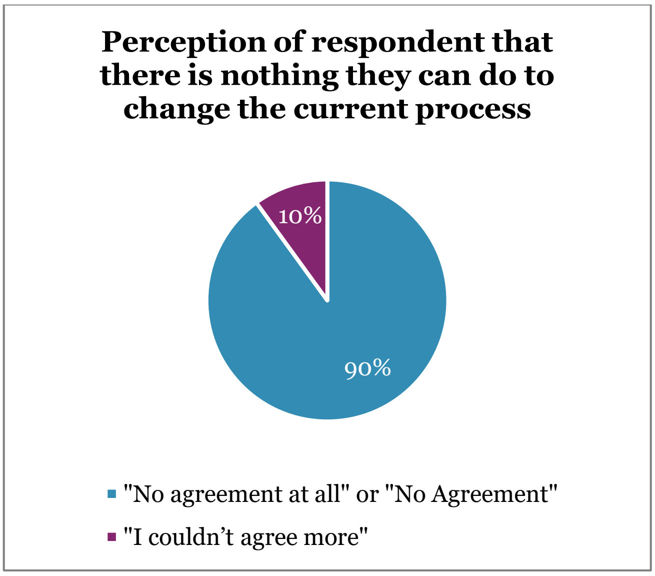

Figure 2 Optimism of respondents for their role in improving surgical data management

CENTRAL DATA MANAGEMENT

Health data management in general is carried out at the DPS at the Bureau d'Information Sanitaire (BIS) via the DHIS2 platform. This is managed at three levels: DSNIS, DPS and health zones, with limited access for DPS and health zones.

Data entry is the task reserved for the health zone via a platform account, and is carried out on a monthly basis until the 20th. The input mask is a photocopy of the various SNIS templates that the Global Fund makes available for each framework.

Programmes not included in the various SNIS templates have additional modules, such as nutrition and epidemiological surveillance. Others, such as the COVID and PNLCMTN campaigns, have accounts created in parallel to the main DHIS2 account for data entry. Only the health zone is authorized to correct an error in the platform. The other two levels extract and analyse data for health and policy decisions.

As far as data is concerned, the system groups the various data elements into Datasets. To see, for example, the surgery data collected by the facilities, consult the Hospital Service Dataset, under the Block Activity heading.

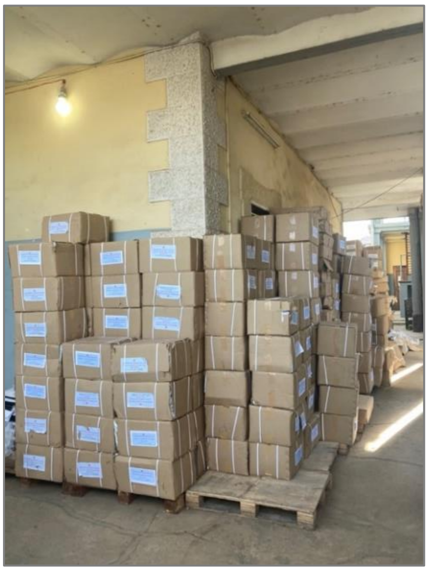

Figure 3 New delivery of data registries from Kinshasa central ministry to Matadi DPS office

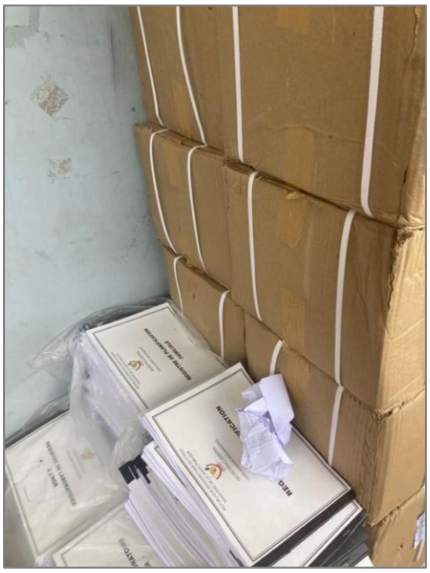

Figure 4 Detail of registries delivered from central Kinshasa ministry

DATA COLLECTED

Whilst there was a common data set present in most theatre logbooks at healthcare facility level, there was also considerable variation. Some healthcare facilities had central government issued theatre registry books, but most facilities had created their own using simple notebooks.

Data present in theatre registry logbooks	Additional data present in some logbooks
• Age<br>• Gender<br>• Date<br>• Diagnosis<br>• Procedure<br>• Infection Y/N<br>• Complications Y/N	• Blood transfusion<br>• Post operative diagnosis<br>• Surgeon<br>• Anaesthetist<br>• Type of anaesthetic

Table 5 Data points in facility level theatre logbooks

Most healthcare facilities do not have unique patient identifier numbers. In addition to this, many patients do not know their exact date of birth. For this reason, it is difficult to follow through individual cases in logbooks. This also makes it difficult to fully track certain indicators such as 30 day mortality, complications and post operative infections.

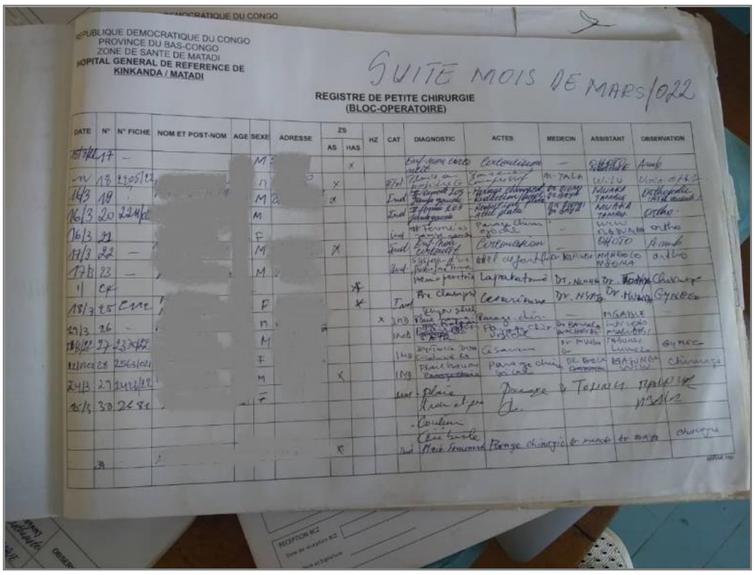

Figure 5 Surgery register/ Theatre logbook

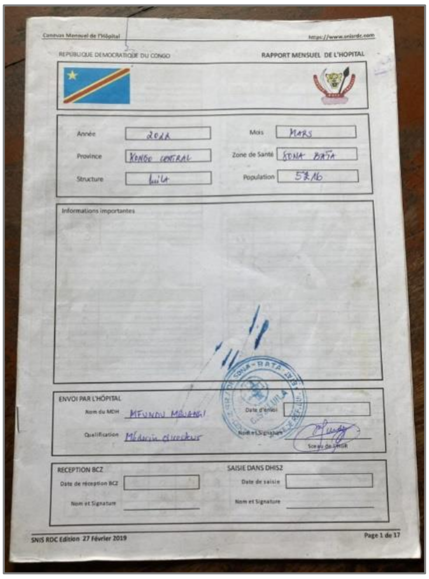

Figure 6 Monthly report from the hospital to the DPS; SNIS

NATIONAL SYSTEM FOR HEALTHCARE INFORMATION/ SYSTÈME NATIONAL D'INFORMATION SANITAIRE (SNIS)

The SNIS is an organised set of structures, institutions, personnel, methods, tools and equipment providing the information required for decision-making, action and management of health programs and systems at all levels (central, intermediate and peripheral).

The DRC SNIS is in widespread use across the country and is the main mechanism by which data is collected at health facility and community level to be sent to provincial level and onwards at national level. Each health zone is responsible for the data it submits at provincial level.

**Data related to surgery in the SNIS<br>(SNIS RDC Edition 27 February 2019)**
Page 10<br>• 6.2 Emergency consultations:<br>➢ Acute surgical abdomen<br>➢ Burns<br>➢ Road traffic collisions<br>➢ Shock<br><br>Page 12<br>• 9.3 Morbidity in surgery<br>• 9.4 Morbidity in obstetrics and gynaecology<br><br>Page 13<br>• 9.6 Death rate registered in hospital<br>• 10.2 Types of obstetrics and gynaecology interventions<br><br>Page 14<br>• 11.1 Type of intervention (major)<br>• 11.2 Type intervention (minor)<br>• 11.3 Functioning of the theatre bloc

Table 6 Detail of SOTA data points in the SNIS currently in use

DISTRICT HEALTH INFORMATION SOFTWARE 2 (DHIS2)

DHIS2 is a tool for collection, validation, analysis, and presentation of aggregate and patient-based statistical data, tailored (but not limited) to integrated health information management activities[12].

Data related to SOTA care is collected on the DHIS2 platform but is not easily searchable in one place. Entry into the DHIS2 platform is dependent on data fields present in the SNIS which do not map exactly. The images below show data fields related to surgical conditions on the DHIS2 platform.

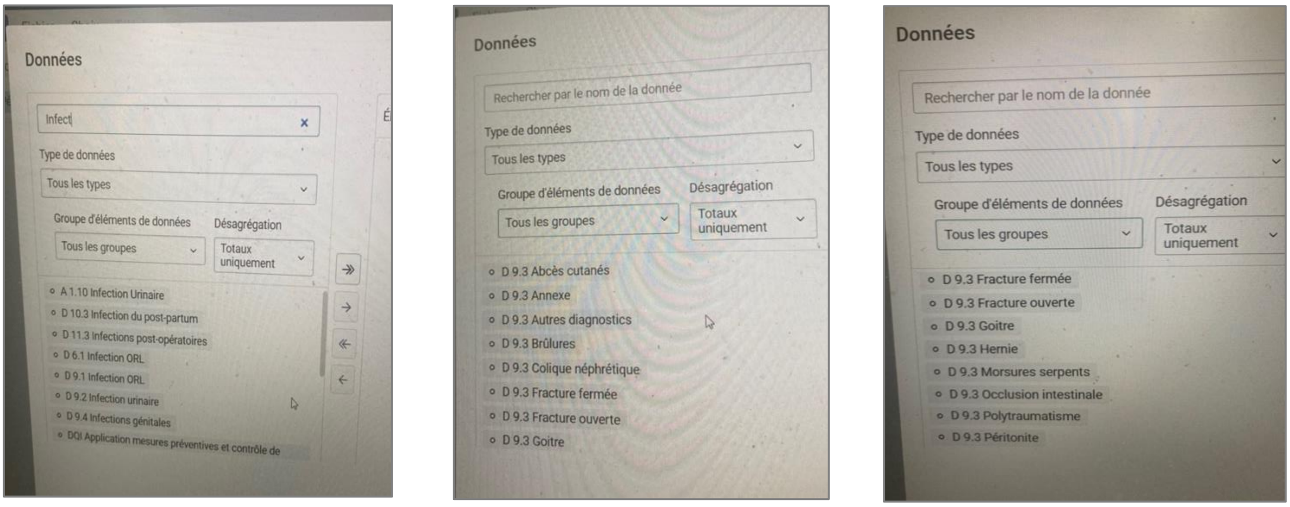

Figure 7 Screenshots showing data fields related to surgical conditions on the DHIS2 platform

DATA COMPLETENESS AND ACCURACY

There are three main sources of SOTA data in Kongo Central (theatre logbooks, SNIS and DHIS2) with multiple steps in data input and transfer. With multiple points of entry for source data and multiple steps in data transfer, the risk of error is high.

Source data into theatre and ward logbooks is done with pen and paper and relies on legible writing, contemporaneous records and an understanding of the clinical details being input. There is a risk here for incomplete and inaccurate data.

Data that is entered into the SNIS relies on source data in logbooks. Data entry may not always be legible and has to be transcribed from one sheet to another. Although there are notes on data entry for the SNIS, some data fields combine multiple data points and some indicators may be entered in more than one place. In addition to this, definitions are not always clear and in the absence of good training and quality control, may be open to interpretation (for example, post operative infection).

Opinions about the quality and accessibility of surgical data was mixed. This is illustrated in Figure 8. 30% of respondents to the survey were of the opinion that data was not easily accessible. The majority of respondents (60%) had a neutral or negative opinion about the quality of data.

[12] DHIS2 https://docs.dhis2.org/en/use/what-is-dhis2.html

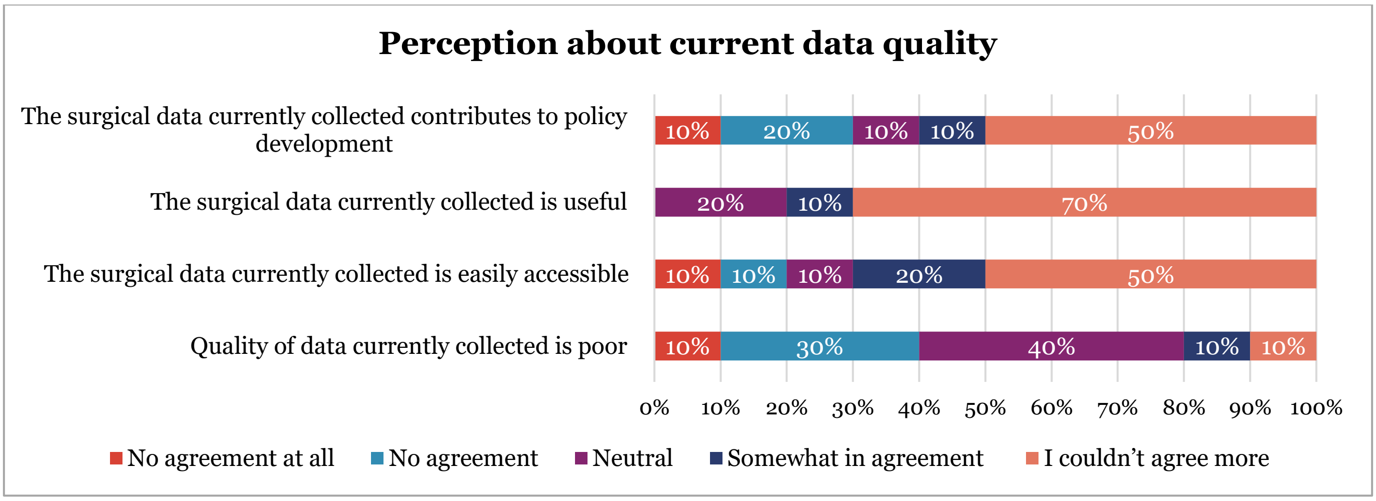

Figure 8 Perception of respondents on current data quality for surgical data

A representative sample from three months of theatre logbook data from the eight healthcare facilities (March, April, May 2022) were analysed for completeness and accuracy of reporting. Results from this are summarised in Table 7.

Healthcare facility	Total number of cases in 3 month period	Percentage missing demographic data (age and/ or gender)	Record of mortality, complications and infection
HPR Kinkanda	157	1.27%	1 case no data entry<br>0 deaths<br>0 infection<br>0 complication
HG Kiamvu	54	23.14%	0 deaths<br>0 infection<br>0 complication
HG Boma	179	1.96%	1 case no data entry<br>2 deaths<br>1 infection<br>1 complication
CSR Kalamu	2	50%	0 deaths<br>0 infection<br>0 complication
IME Kimpese	296	2.19%	0 deaths<br>0 infection<br>0 complication
CS Kimpese	6	33.33%	0 deaths<br>0 infection<br>0 complication
St Luc Kisantu	444	2.58%	0 deaths<br>0 infection<br>0 complication
CSR Luila	26	0%	0 deaths<br>1 infection<br>1 complication

Table 7 Sample analysis of data completeness and accuracy for patient demographics and complications for 8 health facilities

It is worth noting that there is significant variation in the surgical volume between different healthcare faclities but that even sites with small volumes had significant gaps in data completeness. The most striking finding is the lack of accurate reporting on peri-operative mortality and complications, including infection. Only HG Boma and CSR Luila reported any complications, and these are still likely to represent under-reporting. It is unlikely that the reports of no complications for healthcare facilities with high volumes are accurate.

TRIANGULATION THEATRE LOGBOOKS/ SNIS/ DHIS2

To fully understand the correlation of the three data sources, an analysis was done at the ten healthcare facilities in July 2023. This was done as part of the project quarterly visit when seven surgical indicators had been agreed for

collection at facility level. Data for the seven surgical indicators was collected from theatre and ward logbooks, from the SNIS and from DHIS2. These three data sources were then cross-referenced.

This triangulation process revealed significant discrepancies between theatre logbooks, SNIS and DHIS2. This is summarised in Table 8.

Data element	Healthcare facility	Month	Theatre logbook	SNIS framework	DHIS2
Number of major cases	CSR Luila	June 2023	14	18	18
Number of post-op infections	CSR Luila	May 2023	0	1	1
Total number of cases	CSR Luila	May 2023	20	40	27
Number of major cases	CSR Luila	April 2023	4	6	6

Table 8 Triangulation of logbook, SNIS and DHIS2 data

HEALTHCARE FACILITY LEVEL DATA PROCESSES

Each healthcare facility follows a broadly similar process for collecting and evaluating data, although there is some variation in the detail and larger healthcare facilities have more complex processes for data analysis.

Table 9 summarises the key elements of data management at each site.

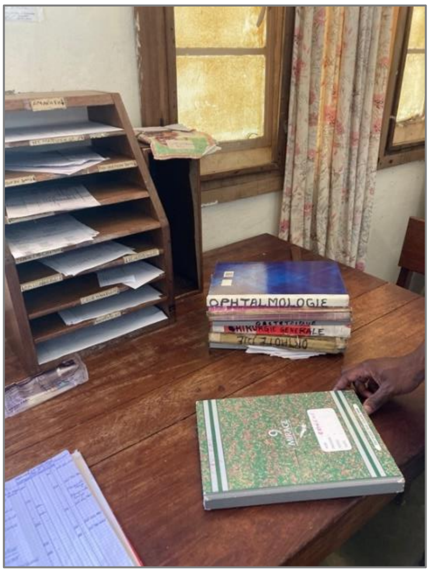

Figure 9 Table in theatre office with multiple theatre logbooks, one for each subspecialty and one overall registry

Healthcare facility	Data collection	Data validation	Persons involved
HPR Kinkanda	Daily in theatre and ward logbooks<br>Monthly into SNIS	Monthly meeting of hospital data committee<br>Direct entry into DHIS2	Nurse in charge theatres<br>Nurse in charge surgical ward<br>Hospital administrator<br>Hospital data committee
HG Kiamvu	Daily in theatre and ward logbooks<br>Monthly into SNIS	Monthly meeting staff including medical director	Nurse in charge theatres<br>Nurse in charge surgical ward<br>Hospital administrator
HG Boma	Daily in theatre and ward logbooks<br>Monthly into SNIS	Monthly meeting of hospital data committee	Nurse in charge theatres<br>Nurse in charge surgical ward<br>Hospital administrator
CSR Kalamu	Daily in theatre and ward logbooks<br>Monthly into SNIS	Monthly meeting staff including medical director	Nurse in charge theatres<br>Nurse in charge surgical ward<br>Hospital administrator
IME Kimpese	Daily in theatre and ward logbooks<br>Monthly into SNIS	Monthly meeting of hospital data committee	Nurse in charge theatres<br>Nurse in charge surgical ward<br>Hospital data team
CS Kimpese	Daily in theatre and ward logbooks<br>Monthly into SNIS	Monthly meeting staff including medical director	Nurse in charge theatres<br>Nurse in charge surgical ward<br>Medical director
St Luc Kisantu	Daily in theatre and ward logbooks<br>Monthly into SNIS	Monthly meeting of hospital data committee	Nurse in charge theatres<br>Nurse in charge surgical ward<br>Hospital data team

Healthcare facility	Data collection	Data validation	Persons involved
CSR Luila	Daily in theatre and ward logbooks<br>Monthly into SNIS	Monthly meeting staff including medical director	Nurse in charge theatres<br>Nurse in charge surgical ward<br>Medical director

Table 9 SOTA data management at healthcare facility level

DATA FLOW

Table 10 summarises the way data is managed across the province with inputs, processing and outputs. It highlights that there is a relatively standardised process for healthcare data management in the province although there is not a specific process that allows for good evaluation and use of SOTA data.

Inputs			Process			Outputs
Action	By whom	Frequency	Action	By whom	Frequency	
Register theatre case in theatre logbook	Surgeon, doctor or nurse	Daily	Data review- surgical department and SNIS	Healthcare data committee at facility level	Monthly	SNIS data for each healthcare facility
Complete SNIS surgery data sections on paper forms	Nurse in charge for each department: surgery, theatres, emergency department	Monthly	Data from paper SNIS book entered into online DHIS2 database	Data officer at facility (tertiary centres) or health zone (health centres)	Monthly	
Collection of different sections of SNIS and collation into single report	Nurse in charge and/ or data officer	Monthly	Verification and correction of data	Healthcare data committee at facility level	Monthly	
Data from facility level on SNIS and/ or DHIS2		Monthly	Review of SNIS facility level data	DPS	Monthly	

Table 10 Data flow in the Kongo Central Province

USE OF DATA FOR POLICY

Most respondents fully agree that the data collected on surgery is useful, although there is a lack of agreement about the quality of collected data. As shown in Figure 8 on page 12, 60% respondents believe that the data collected contributes to policy development. 70% respondents agree that the data is easily accessible.

Summary of findings

This situational analysis describes the current flow of health care data- particularly related to SOTA care – in the Kongo Central province. Key findings include:

- A lack of awareness amongst healthcare workers, administrators and policy makers of recommended surgical indicators.
- A heterogenous process for data collection, analysis and use across healthcare facilities, health zones and provincial leadership.
- There is disconcordance between different data sets used to monitor surgical activity and indicators.

- There is a willingness to improve processes for surgical data collection in the province and this may be used as an example for national scale up.

Recommendations

This work identified key areas to implement change and we make the following recommendations:

- Surgical indicators to considered essential in the monitoring and evaluation of healthcare delivery in the province.
- The 7 new surgical indicators should be collected at all healthcare facilities as part of the monthly healthcare data collection and analysis process, led by the DPS.
- Support initiatives, including further training, to improve the concordance of data between the three data sources.
- The surgical indicators should be used to inform healthcare policy.
- At project end (February 2024), a thorough analysis of the impact of the implemented changes should take place.
- Findings from this work should be presented to all stakeholders, and senior health care leaders at national level for potential national roll out.

KING'S COLLEGE LONDON
Centre for Global Health and Health Partnerships
6th floor, Addison House, Guy's Campus, Henriette Raphael Building, London SE1 1UL

T: 020 7848 5060 **W:** kcl.ac.uk/KGHP
E: kghp@kcl.ac.uk @KingsGHP
