# Supplementary material for: Health systems strengthening in the Democratic Republic of Congo: the importance of surgical data
Source: BMJ Glob Health. 2025 Sep 4;10(9):e017759. doi: 10.1136/bmjgh-2024-017759 (PMC12414223; doi:10.1136/bmjgh-2024-017759)
Supplement: online supplemental file 6 [file bmjgh-10-9-s006.pdf]

Registre d’activite chirurgicale RDC Kongo Central Hopital

Numéro de patient	Âge (années)	Date de la chirurgie	Durée du séjour (jours)	Diagnostic	Intervention	Check-list de l’OMS utilise? (Oui / Non)	Réopération dans 30 jours (Oui /Non)	Décédé (Oui /Non)	Date de dece	Complications (veuillez énumérer)	Type d’anesthesie	Infection postopératoire (Oui /Non)

King’s Global Health Partnerships

MINISTERE DE LA santé

KING'S HEALTH PARTNERS

KING'S College LONDON

Registre d’activite chirurgicale RDC Kongo Central
Hopital

Registre d’activite chirurgicale RDC Kongo Central: Notes pour remplir le canevas

Cette feuille sert à enregistrer l'activité chirurgicale dans votre hôpital ou centre de santé. Elle est conforme aux directives de l'OMS sur la collecte de données chirurgicales et aux recommandations des autorités provinciales.

Veuillez noter les points importants suivants pour remplir cette fiche.

Chaque intervention dans le bloc opératoire doit être enregistrée. Cela comprend les cas d'urgence et les cas programmees, les cas majeurs et les cas mineurs.

Une partie de la feuille doit être remplie par l'équipe du bloc opératoire et une autre par l'équipe du pavilion.

La collecte des données doit se faire en équipe, mais la responsabilité apartient au chef du service de chirurgie. Chaque site peut adapter sa méthode de collecte de données en fonction de sa configuration.

Les données doivent être envoyées au DPS à la fin de chaque mois, en même temps que la collecte mensuelle standard.

Si vous avez des questions ou si vous avez besoin de conseils, veuillez contacter votre chef de service de chirurgie ou le point focal Chirurgie en securite. Si vous avez des commentaires sur le formulaire ou le processus, veuillez contacter le Dr Achim Mambu Vangu.
